# Guiling prescription attenuates hyperuricemia via multi-target regulation of uric acid metabolism, renal protection, and inflammation: insights from metabolomics and network pharmacology

**DOI:** 10.3389/fnut.2025.1738623

**Published:** 2026-01-26

**Authors:** YuKun Wang, RenJie Ding, Yaxuan Guo, TianHui Zhou, Huichun Zhao, HuiWu Liu, XueMei Qin, XiaoXia Gao

**Affiliations:** 1 Modern Research Center for Traditional Chinese Medicine, Shanxi University, Taiyuan, Shanxi, China; 2 The Key Laboratory of Chemical Biology and Molecular Engineering of Ministry of Education, Shanxi University, Taiyuan, Shanxi, China; 3 Key Laboratory of Effective Substances Research and Utilization in Traditional Chinese Medicine (TCM) of Shanxi Province, Shanxi University, Taiyuan, Shanxi, China; 4 Shanxi Guangyuyuan Traditional Chinese Medicine, Jinzhong, Shanxi, China

**Keywords:** guiling prescription, hyperuricemia, metabolomics, network pharmacology, uric acid excretion, uric acid synthesis

## Abstract

**Background:**

This study aims to evaluate the efficacy of Guiling Prescription (GP)—a medicinal food homologous formula—in hyperuricemic rats, its effects on uric acid excretion and renal function, and to clarify the metabolic mechanisms involved in GP's alleviation of hyperuricemia.

**Methods:**

Sprague-Dawley (SD) rats of hyperuricemia was established using potassium oxonate (200 mg/kg, PO) and adenine (100 mg/kg) to assess the therapeutic effects of Guiling Prescription (GP). We measured body weight, serum levels of uric acid and creatinine, as well as xanthine oxidase (XOD) and adenosine deaminase (ADA) activity, alongside histopathological parameters. Serum concentrations of interleukin-6 (IL-6), tumor necrosis factor-α (TNF-α) and alanine aminotransferase (ALT), and aspartate aminotransferase (AST) were determined using ELISA kits. The expression of renal uric acid transporters was evaluated through Western blotting. Network pharmacology was utilized to predict the key drug-disease targets, and a non-targeted metabolomic assay was applied to identify the key metabolites and metabolic pathways, and validated these targets through molecular docking and western blot analyses.

**Results:**

GP showed an improvement effect on hyperuricemia model rats, with decreased levels of serum uric acid (UA), serum urea nitrogen, and creatinine, and serum ALT, AST. Furthermore, H&E staining results showed to improve renal injury in the hyperuricemic rat, and serum interleukin-6 and tumor necrosis factor-αwere improve the body's inflammatory response after administration of GP. In addition, GP could regulate multiple serum metabolic pathways such as arachidonic acid metabolism, pyrimidine metabolism, purine metabolism, citric acid cycle. On one side, GP decreased the synthesis of uric acid by inhibiting hepatic xanthine oxidase activities and adenosine deaminase activity. On the other side, GP increased the excretion of uric acid with the upregulation of UA excretion genes ABCG2, OAT1, and OAT3 and downregulation of UA resorption genes URAT1 and GLUT9.

**Conclusion:**

GP orchestrates uric acid metabolism through multi-target and multi-pathway regulation, highlighting its potential not only as a novel therapeutic strategy but also as a promising dietary supplement for the management of hyperuricemia.

## 1 Introduction

For Hyperuricemia (HUA), a chronic metabolic disorder caused by dysregulated purine metabolism and impaired uric acid (UA) excretion (1), has seen a significant rise in global prevalence due to modern lifestyle and dietary changes (2). Beyond its direct health impacts, HUA is closely associated with metabolic syndrome (3), which increases the risk of cardiovascular diseases, diabetes, and chronic kidney injury (4, 5). Current therapeutic strategies, such as uricosuric agents (e.g., benzbromarone) and xanthine oxidase inhibitors (e.g., allopurinol), are limited by side effects and transient efficacy, failing to address the multifactorial pathogenesis of HUA (6). Consequently, the growing burden of HUA necessitates innovative and safer interventions. Recent studies have highlighted the potential of natural compounds derived from herbal medicines, which exhibit multi-target mechanisms, complex interactions, and low toxicity and side effects, in reducing UA levels (7). These properties offer a novel strategy for managing HUA, providing a promising alternative to conventional therapies.

Guiling Prescription (GP) is a modernized traditional Chinese medicine (TCM) formula derived from classical prescriptions through strategic herbal modifications, emphasizing spleen qi tonification, liver-kidney nourishment, UA excretion promotion, and qi regulation. Through multi-target synergistic effects, GP comprehensively regulates metabolic functions in the body (8). This formula has obtained a production license in China (Shanxi: SC12714072602558) and a national authorized invention patent (Patent Number: ZL 202210170032.0). GP consists of nine high-quality medicinal herbs, including spleen qi-tonifying *Panax ginseng* C. A. Mey., *Poria cocos* (Schw.) Wolf, *Lilium pumilum* DC., and *Ziziphus jujuba* Mill.; liver-kidney-nourishing *Lycium barbarum* L., *Polygonatum sibiricum* Red., and *Dioscorea opposita* Thunb.; and heat-clearing and blood-activating *Pueraria lobata* (Willd.) Ohwi and *Perilla frutescens* (L.) Britt. All plant names were validated using the World Flora Online (http://www.theplantlist.org). From the perspective of TCM theory and principles, GP was considered to have safely and effectively alleviated HUA. Additionally, modern studies have demonstrated that the constituent herbs of GP, such as *Panax ginseng* C.A.Mey (9), *Perilla frutescens* (L.) Britt. (10), *Dioscorea opposita* Thunb. (11), *Pueraria lobata* (Willd.) Ohwi. (12), *Lycium barbarum* L. (13), exhibit significant urate-lowering effects. Key active components in GP, including puerarin, pueraria glycosides (14), scutellarein (15), ligustrazine (16), berberine (17), apigenin (18), and ginsenosides (19), can significantly relieve HUA and inflammation. These findings indicate that GP shows significant promise in treating HUA. Notably, all nine herbal components of GP are officially listed as both medicinal and edible substances in China, embodying the principle of medicinal food homology. This intrinsic characteristic suggests a high safety profile and positions GP not merely as a therapeutic agent, but as a promising candidate for long-term dietary-like intervention in hyperuricemia. Despite its notable clinical efficacy, further investigation is imperative to explore its underlying mechanisms.

The rapid evolution of artificial intelligence-driven models has significantly advanced the application of network pharmacology in deciphering the complex mechanisms of TCM for metabolic disorders (20–22). Concurrently, metabolomics provides a holistic approach to characterize metabolite-pathology relationships through systematic quantification of endogenous small molecules (23). While network pharmacology facilitates multi-target prediction, it often lacks experimental validation; metabolomics, though robust in profiling metabolic shifts, faces limitations in identifying therapeutic targets and pathways. Integrating these methodologies offers synergistic advantages, bridging hypothesis generation with mechanistic validation. Building on this paradigm, our study combines LC-MS-based metabolomics and network pharmacology to elucidate the metabolic mechanisms and therapeutic targets of GP in HUA.

In this study, we evaluated the therapeutic efficacy of GP using a potassium oxonate (PO) and adenine induced HUA rat model through integrated pharmacodynamic, metabolomic, and network pharmacology analyses. GP was systematically compared with three positive control drugs: benzbromarone (a uricosuric agent), allopurinol (a xanthine oxidase inhibitor), and Simiao pill (a urate-lowering TCM formula). Comprehensive analysis of physiological, pathological, and metabolomic data revealed that GP not only restored serum uric acid (SUA) levels but also modulated key renal transport proteins and suppressed inflammatory pathways. Network pharmacology further uncovered GP's multi-target mechanisms, particularly its regulation of purine metabolism and TNF signaling. By combining experimental validation with computational prediction, this study elucidated the therapeutic efficacy of GP in managing HUA and clarified its underlying mechanisms of action. These findings establish a foundation for advancing GP as a promising dietary supplement for the management of hyperuricemia.

## 2 Materials and methods

### 2.1 Reagents and chemicals

PO and adenine were purchased from Sangon Biotech Co., Ltd. (Shanghai, China). Kits for measuring SUA, blood urea nitrogen (BUN), and creatinine were sourced from Jiancheng Bioengineering Institute (Nanjing, China). Serum interleukin-6 (IL-6) and tumor necrosis factor-α (TNF-α) kits were obtained from Wuhan GeneCreate Engineering (Wuhan, China). Antibodies for ATP binding cassette subfamily G member 2 (ABCG2), organic anion transporter 1 (OAT1), organic anion transporter 3 (OAT3), urate anion transporter 1 (URAT1), and glucose transporter 9 (GLUT9) were provided by ABclonal (Wuhan, China). Tissue protein extraction kits and the enhanced bicinchoninic acid protein assay were supplied by Boster Biological Technology (China). Benzbromarone was obtained from Excellla GmbH in Nurnberg, Germany. Allopurinol was sourced from Sine Wanxiang Pharmaceutical Co., Ltd. (Shanghai, China). Simiao pill was acquired from Jilin Zixin Pharmaceutical Co., Ltd. based in Jilin, China. High-performance liquid chromatography (HPLC)-grade methanol, formic acid, acetonitrile, propanol, and pure water were obtained from Thermo Fisher Scientific (Waltham, MA, United States).

### 2.2 Sources of herbs in GP

The components of GP with their Chinese pinyin name, Latin name, Chinese Medicine Name and relative ratios in application are shown in Table 1. Samples of the GP were generously supplied by Guangyuyuan Traditional Chinese Medicine Co., Ltd. (Approval number: SC12714072602558, Jinzhong, China).

**Table 1:** The components of GP.

**Pinyin name**	**Latin name**	**Chinese medicine name**	**Amount in application (%)**
Shanyao	*Dioscorea opposita* Thunb.	Dioscoreae Rhizoma	15
Gegen	*Pueraria lobata* (Willd.) Ohwi.	Puerariae Lobatae Radix	15
Huangjing	*Polygonatum kingianum* Coll.et Hemsl.	Polygonati Rhizoma	10
Fuling	*Poria cocos* (Schw.) Wolf.	Poria	10
Baihe	*Lilium lancifolium* Thunb.	Lilii Bulbus	10
Renshen	*Panax ginseng* C. A. Mey.	Ginseng Radix Rhizoma	10
Zisuye	*Perilla frutescens* (L.) Britt.	Perillae Folium	10
Gouqizi	*Lycium barbarum* L.	Lycii Fructus	10
Dazao	*Ziziphus jujuba* Mill.	Jujubae Fructus	10

### 2.3 Chemical composition analysis of GP based on UPLC-Q-TOF-MS analysis

The chemical composition of GP was analyzed using ultra-performance liquid chromatography coupled with time-of-flight mass spectrometry (UPLC-Q-TOF-MS). Detailed conditions for chromatographic separation and mass spectrometry are provided in the “Chemical Composition Analysis of GP Using UPLC-Q-TOF-MS” section of the Supplementary material.

### 2.4 Experimental animals

All male Sprague-Dawley rats, weighing approximately 180 ± 20 g and aged 6 weeks [Registration No. SCXK (JING) 2024-0601], were sourced from the Experimental Animal Center at Weitong Lihua Technology Co. Ltd. (Beijing, China). Before participating in the experiments, the rats underwent a one-week acclimatization period. The animals were housed in a specialized SPF-grade Experimental Animal House that maintained specific environmental conditions, including a temperature range of 22−25 °C, humidity levels of 45−60%, and a 12-h light/dark cycle, with unrestricted access to food and water. All animals received humane care, and the research protocol was approved by the Animal Ethics Committee of Shanxi University (approval number SXULL2023029). This study was conducted in accordance with the ARRIVE (Animal Research: Reporting of *In Vivo* Experiments) guidelines.

### 2.5 Induction of hyperuricemia model and drugs treatment

**Figure 1:**
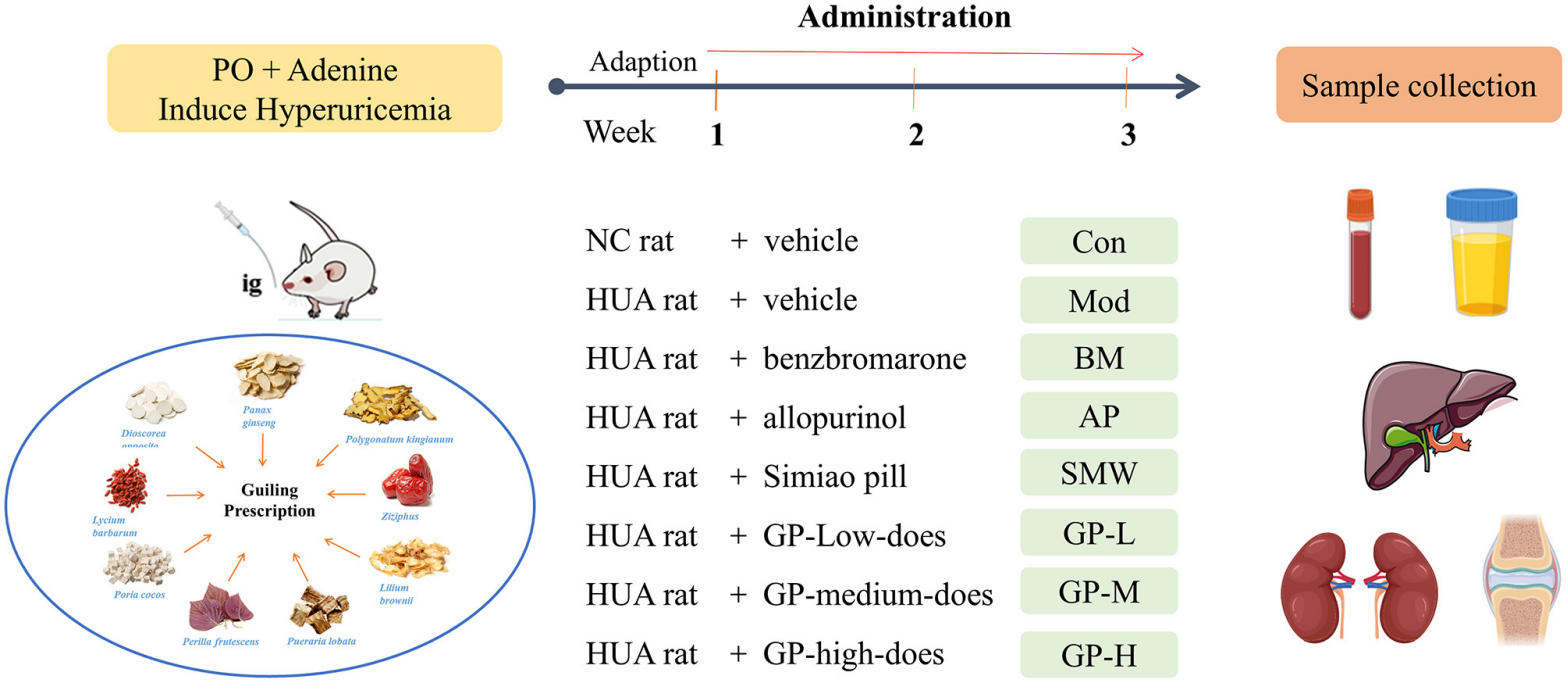
Experimental procedure to study the action of GP on HUA rats.

After a week of adaptive feeding, 64 male rats were randomly assigned to eight groups, each containing eight rats: control group (Con), model group (Mod), three positive control groups (benzbromarone group, BM, 10 mg/kg BW/day; allopurinol group, AP, 20 mg/kg BW/day; Simiao pill group, SMW, 1.2 g/kg BW/day), and three doses of GP groups (high-dose group GP-H, 49.6 g/kg BW/day; medium-dose group GP-M, 24.8 g/kg BW/day; low-dose group GP-L, 12.4 g/kg BW/day). The Con group received normal saline containing 0.5% sodium carboxymethyl cellulose (CMC-Na), while the other groups were administered potassium oxonate (PO, 200 mg/kg) and adenine (100 mg/kg) daily by gavage for 3 weeks to induce hyperuricemia. Both potassium oxonate and adenine were dissolved in 0.5% CMC-Na solution for administration. The experimental protocol is illustrated in Figure 1. On the 21st day of the experiment, the metabolic cages were used to collect the fecal samples (containing urine and fecal-mixture). After 3 weeks, rats were euthanized under deep anesthesia (2–5% isoflurane) via abdominal aorta blood collection and subsequent decapitation, with all efforts made to alleviate suffering. The liver, kidney, and knee joint samples were collected for analysis. The entire left kidney and left knee joint were fixed in 4% paraformaldehyde for histopathological studies, while the remaining samples were stored at −80 °C for further analysis.

### 2.6 Biochemical analysis

Prior to analysis, urine samples underwent 10-fold dilution with normal saline. Fecal specimens were homogenized (1:9 w/v in normal saline) using a mechanical homogenizer for 60 s, followed by centrifugation at 10,000 rpm for 10 min at 4 °C. Liver xanthine oxidase (XOD) and adenosine deaminase (ADA) activities were quantified using commercially available assay kits according to manufacturer protocols (BioVision Inc., USA). UA concentrations in serum, urine, and fecal supernatants were determined through the phosphotungstic acid-reduction method. Serum creatinine (Scr) levels were analyzed via sarcosine oxidase-peroxidase enzymatic assay, while BUN concentrations were measured using urease-glutamate dehydrogenase coupled reactions. Levels of IL-6 and TNF-α, both in serum and joint tissue homogenates, were quantified using commercial enzyme-linked immunosorbent assay (ELISA) kits (Jiancheng Bioengineering Institute, Nanjing, China) according to the manufacturer's protocols. Serum aliquots were analyzed for alanine aminotransferase (ALT), and aspartate aminotransferase (AST) using standardized commercial assay kits per manufacturer protocols (Jiancheng Bioengineering Institute, Nanjing, China). All assays included appropriate quality controls and were performed in strict adherence to the kit instructions to ensure precision.

### 2.7 Histopathological analysis of kidney and knee joint

The kidneys and joints fixed in formalin were processed for paraffin embedding, followed by sectioning and staining with hematoxylin and eosin (H&E) to observe pathological changes. Microscopic examination of H&E-stained sections allowed for the assessment of histological changes, including the accumulation of urate crystals.

### 2.8 Untargeted metabolomics analysis

#### 2.8.1 Pre-processing of serum samples and UHPLC-MS/MS analysis

The serum samples were thawed at 4 °C, and 100 μL was transferred to a 1.5 mL EP tube. Then, 200 μL of 0.1% formic acid in acetonitrile was added, and the mixture was vortexed for 3 min to ensure thorough mixing. In addition, equal volumes of each sample solution were taken separately and mixed to prepare the quality control (QC) sample. Detailed methods and procedures for the metabolomics analyses, including chromatographic separation conditions and mass spectrometry parameters, are provided in the “Metabolomics Analysis Based on UHPLC-Q Exactive Orbitrap-MS” section of the Supplementary material.

#### 2.8.2 Metabolomics data processing and multivariate pattern analysis

For the metabolomics, the LC-MS raw data were exported by Xcalibur workstation (Thermo Fisher Scientific Inc., Waltham, MA, USA), and then Compound Discoverer 3.1 (Thermo Fisher, USA) was used to give information on the matched and aligned peak data. The parameters were under the following settings: mass tolerance: 5 ppm; RT tolerance (min): 0.05; S/N threshold: 3; mass range: 100–1,500 Da; normalize areas: constant mean.

To conduct multivariate statistical analysis, SIMCA-P 13.0 (Umetrics, Sweden) was carried out for the principal components analysis (PCA), partial least-squares discriminant analysis (PLS-DA) and orthogonal partial least-squares discriminate (OPLS-DA) analysis. The variable importance in the projection (VIP) represents the confirmation of the importance of the variables. Metabolites satisfying both conditions of VIP > 1 and *P* < 0.05 were selected as candidate metabolites. All statistically significant differential metabolites were further subjected to the Benjamini–Hochberg procedure to control the False Discovery Rate (FDR). An FDR-adjusted *p*-value (*q*-value) threshold of <0.05 was applied for final metabolite selection. To identify the metabolites and get more knowledge of the metabolic pathways, Human Metabolome Database (HMDB, http://www.hmdb.ca/spectra/ms/search), Kyoto Encyclopedia of Genes and Genomes (KEGG, http://www.genome.jp/kegg/), m/z cloud (https://www.mzcloud.org/), Metabo Analyst 4.0 (http://www.metaboanalyst.ca/) were applied.

### 2.9 Network pharmacology analysis

The main chemical constituents of GP were identified using UPLC-Q-TOF-MS technology, and their Canonical SMILES were retrieved from the PubChem database (https://pubchem.ncbi.nlm.nih.gov/). Potential targets of these constituents were predicted using the SwissTarget database (http://swisstargetprediction.ch/), TCMSP (https://www.tcmsp-e.com/), HERB (http://herb.ac.cn/), and ETCM (http://www.tcmip.cn/ETCM2/front/#/). The term “hyperuricemia” was searched in GeneCards (https://www.genecards.org/), OMIM (https://www.omim.org/), TTD (http://db.idrblab.net/ttd/), and DisGeNET (https://www.disgenet.org/) databases to identify HUA-related therapeutic targets. The HUA targets were mapped with the drug action targets to obtain the intersection targets and displayed them using a Venn plot. Then, the “Traditional Chinese Medicine-Ingredient-Target” network diagram was constructed by Cytoscape 3.10.2 software (24). To investigate the biological process of HUA further, we used the Metascape database (https://metascape.org/) for Gene Ontology (GO) and the Kyoto Encyclopedia of Genes and Genomes (KEGG) study. All data were uploaded to the bioinformatics platform (http://www.bioinformatics.com.cn/) for display and analysis.

### 2.10 Molecular docking

The crystal structure of the protein target was obtained from the Protein Data Bank (PDB, https://www.rcsb.org/) database. The three-dimensional ligand structure was acquired from the PubChem (https://pubchem.ncbi.nlm.nih.gov/) database. Molecular docking was carried out utilizing the CB-dock2 online platform (https://cadd.labshare.cn/cb-dock2/index.php) (25).

### 2.11 Western blotting

Western blotting was used to evaluate the expression levels of ABCG2, OAT1, OAT3, URAT1, and GLUT9 *in vivo*. Total protein was extracted from kidney samples using 10% (w/v) RIPA Lysis Buffer. Subsequently, the lysates were subjected to centrifugation for a period of 15 min (4 °C, 12,000 rpm). The concentration of extracted protein was then determined using the bicinchoninic acid assay. A total of 20 μg of protein was subjected to SDS-PAGE separation and subsequently transferred to a PVDF membrane. The PVDF membrane was initially blocked with 5% skim milk for 1 h, after which it was incubated with the corresponding primary antibody at 4 °C overnight. The PVDF membrane was subjected to three washes with PBST, following which it was incubated with horseradish peroxidase (HRP)-conjugated secondary antibody for 1 h at room temperature. Thereafter, the bands were visualized by means of an enhanced chemiluminescence reagent (Meilun, Dalian, China). Kidney tissue samples for Western blot analysis were randomly selected from each treatment group, with priority given to samples that exhibited median values in key biochemical parameters (serum uric acid and creatinine) to best represent the group's overall response.

### 2.12 Statistical analysis

Results were expressed as mean ± SEM. Intergroup differences in the biochemical analysis were carried out by analysis of variance (ANOVA) model with SPSS software (version 22.0; SPSS Inc., Chicago, IL, USA), and *P*-values less than 0.05 were considered statistically significant.

## 3 Results

### 3.1 Characterization of chemical components of GP based on UPLC-Q-TOF-MS

The UPLC-Q-TOF-MS data was used for the identification of compounds in the GP firstly (Supplementary Figure S1). A total of 124 compounds were identified by comparing the mass data with the corresponding standards or mass data from the literature (Supplementary Table S1). Among these, saponins and flavonoids were the primary bioactive components, such as Puerarin, Pueraria glycoside (14), Baicalin (15), Ligustrazine (16), Luteolin (26), berberine (17), Apigenin (18), and Ginsenosides (19).

### 3.2 GP reduces UA levels in rats by modulating UA synthesis and excretion

**Figure 2:**
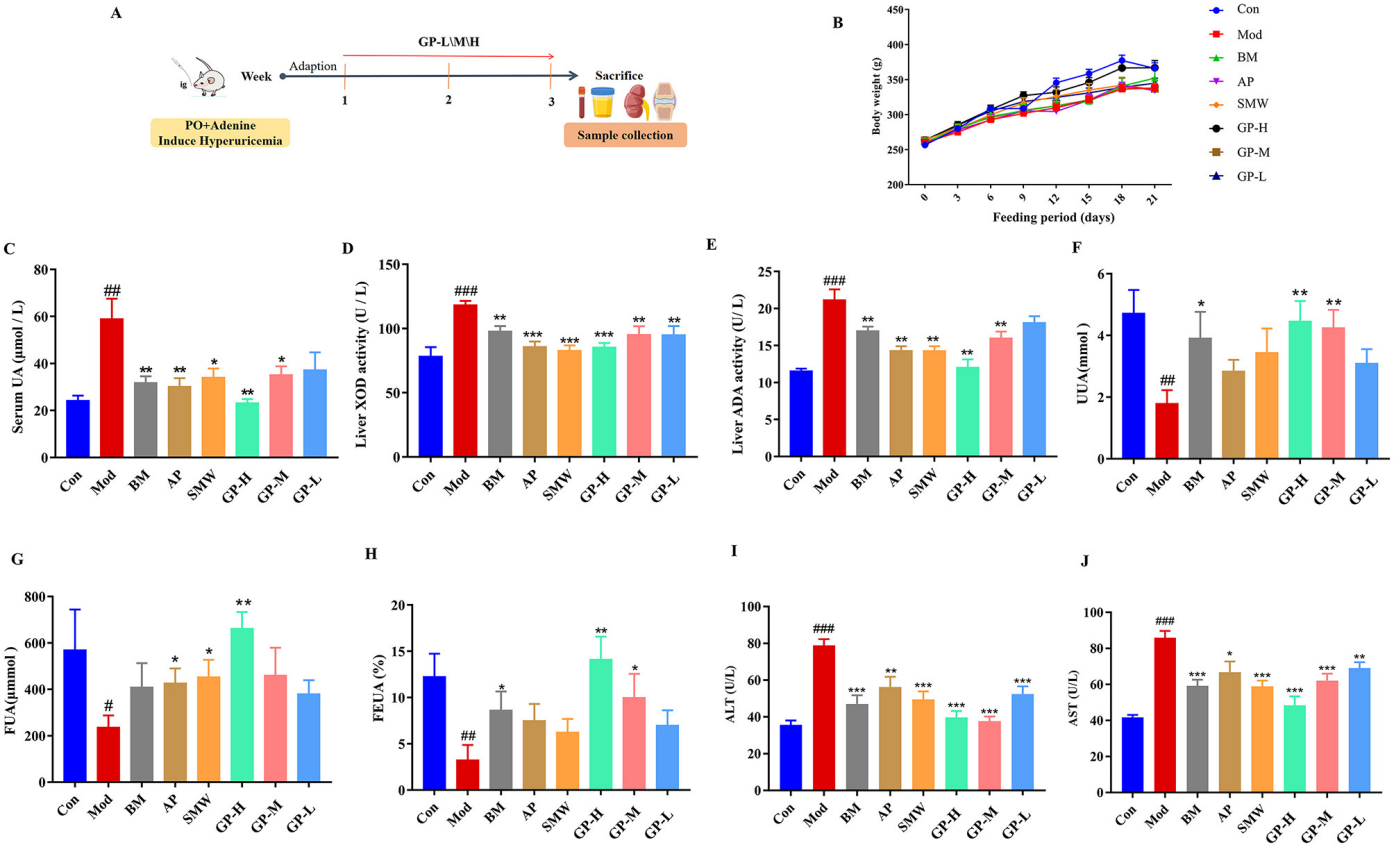
Effects of GP on HUA rats induced by oral administration of PO and adenine. **(A)** Experimental design procedure. **(B)** Body weight changing curve. **(C)** SUA. **(D)** Liver XOD activity. **(E)** Liver ADA activity. **(F)** Urine UA. **(G)** Feces UA. **(H)** FEUA. **(I)** ALT. **(J)** AST. Data are presented as mean ± S.E.M (*n* = 8). ^#^*P* < 0.05, ^##^*P* < 0.01, ^###^*P* < 0.001 vs. Con; *P < 0.05, **P < 0.01, ***P < 0.001 vs. Mod.

During the 21-day animal experiment (Figure 2A), the body weight of rats in each group was measured every 3 days (Figure 2B). The results demonstrated that the Mod group exhibited a lower body weight compared to the Con group. And the GP-H treatment group showed a tendency toward weight recovery.

SUA is a primary clinical diagnostic marker for HUA. To further evaluate the efficacy of the HUA model and the therapeutic effects of positive control drugs and GP, we measured the SUA levels in rats. As shown in Figure 2C, after 3 weeks of continuous administration, the SUA levels in the Mod group increased by 242.9% compared to the Con group, confirming the successful establishment of the HUA model. The SUA levels in all three positive treatment groups were significantly reduced: AP group (30.2 μmol/L, *P* < 0.01), BM group (31.8 μmol/L, *P* < 0.01), and SMW group (34.1 μmol/L, *P* < 0.05), indicating that these drugs effectively alleviated HUA. Furthermore, the GP-H and GP-M groups exhibited significant reductions in SUA levels by 60.7% and 40.3%, respectively, demonstrating the potential of GP to mitigate HUA. Notably, GP-H outperformed benzbromarone, allopurinol, and Simiao pill in alleviating HUA in rats.

The measurement of XOD and ADA activities revealed that, after 3 weeks of treatment, the Mod group exhibited significantly elevated hepatic XOD (*P* < 0.001) and ADA (*P* < 0.001) activities compared to the Con group, confirming the successful establishment of HUA (Figures 2D, E). In contrast, all drug treatment groups significantly reduced hepatic XOD and ADA activities. Notably, the GP-H group demonstrated a marked downregulation of XOD (*P* < 0.001) and ADA (*P* < 0.01) activities, with GP showing a dose-dependent effect, particularly on ADA activity. These findings suggest that the ability of GP to reduce UA levels may be associated with its inhibitory effects on hepatic XOD and ADA activities.

Furthermore, we investigated the effect of GP on UA excretion by measuring 12-h urinary and fecal UA excretion. As shown in Figures 2F, G, compared to the Con group, the Mod group exhibited a significant reduction in urinary UA excretion (*P* < 0.01) and fecal UA excretion (*P* < 0.05). Notably, the positive drug benzbromarone significantly increased urinary UA excretion (*P* < 0.05), while allopurinol and Simiao pill significantly enhanced fecal UA excretion (*P* < 0.05). Interestingly, the GP-H group demonstrated a marked increase in both urinary UA (*P* < 0.01) and fecal UA (*P* < 0.01) excretion, showing broader therapeutic efficacy compared to the positive drugs. We also calculated the fraction excretion of uric acid (FEUA). Compared to the Mod group, benzbromarone, allopurinol, and Simiao pill improved FEUA, while GP exhibited a more pronounced enhancement in FEUA than the three positive drugs (Figure 2H). These results clearly indicate that GP effectively regulates UA clearance in HUA rats by enhancing UA excretion through both renal and intestinal pathways, demonstrating superior efficacy compared to the positive drugs.

To preliminarily assess the hepatic safety profile of GP, we measured serum ALT and AST, the most sensitive and established biomarkers for drug-induced liver injury. As expected, the Model group exhibited significantly elevated levels of both ALT and AST compared to the Control group, reflecting metabolic stress associated with HUA. Importantly, GP treatment did not induce any significant increase in either transaminase compared to the Control group, and their levels were significantly lower than those in the Model group (Figures 2I, J). These results indicate that GP, even at its highest efficacious dose, did not elicit hepatotoxicity during the 3-week treatment period. Notably, while the positive control drug allopurinol effectively lowered uric acid, it is associated with potential hepatic adverse effects. In contrast, GP demonstrated comparable urate-lowering efficacy without elevating liver injury markers in this model, highlighting its favorable safety profile and underscoring a potential advantage of multi-component herbal formulations in minimizing target organ toxicity.

### 3.3 GP ameliorates renal injury in HUA rats by regulating Scr and BUN levels

**Figure 3:**
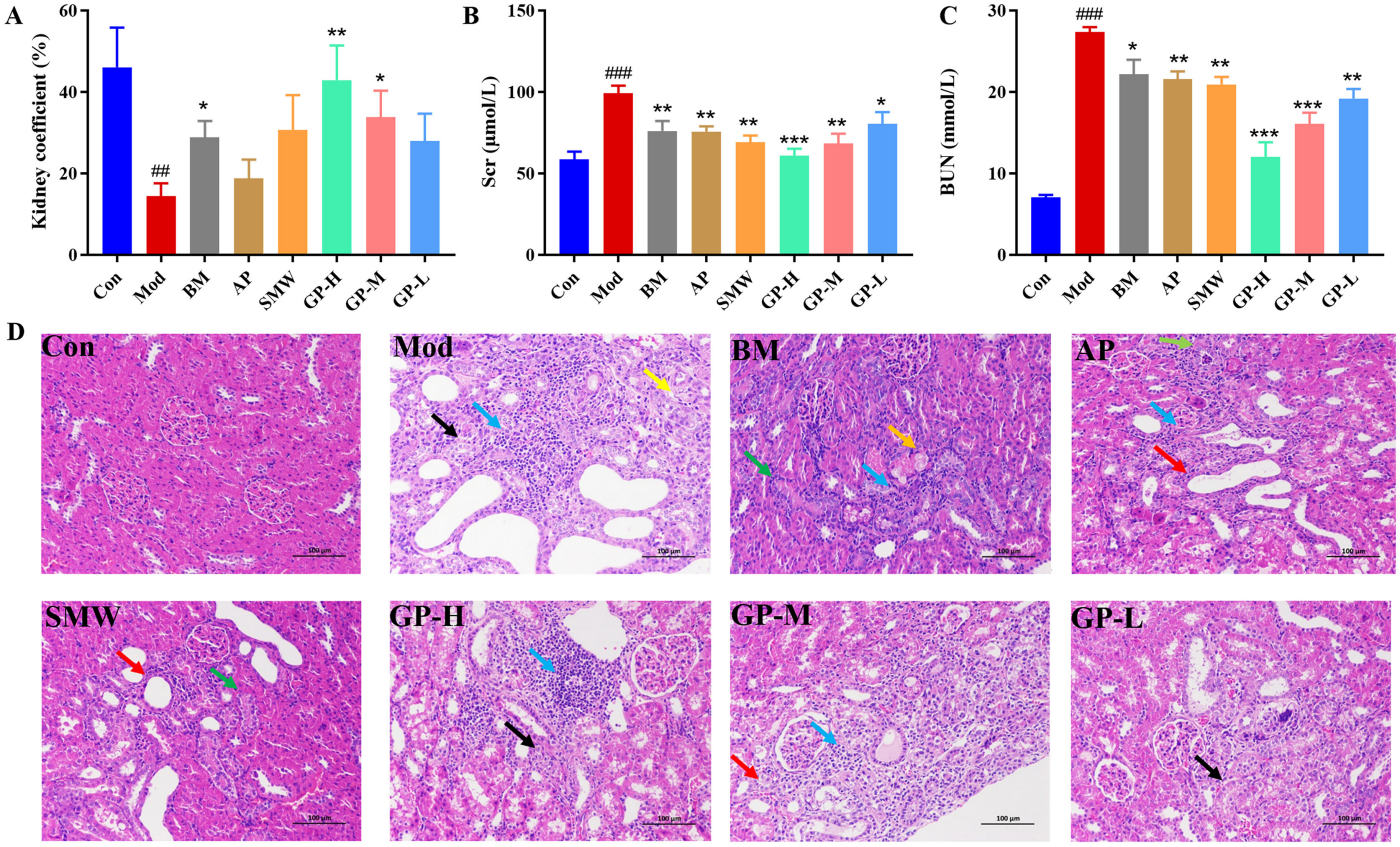
GP ameliorates renal injury in HUA rats by regulating creatinine and BUN. **(A)** Renal organ index. **(B)** Scr. **(C)** BUN. **(D)** Ameliorative effect of GP on kidney tissue pathological alterations in HUA rats (HE staining images of kidney sections at magnification 200×). Black arrows: loose renal tubular cells; yellow arrows: interstitial connective tissue hyperplasia; red arrows: tubular dilation; blue arrows: lymphocyte infiltration; green arrows: tubular atrophy; orange arrows: urate deposition. Data are presented as mean ± S.E.M (*n* = 8). ^##^*P* < 0.01, ^###^*P* < 0.001 vs. Con; *P < 0.05, **P < 0.01, ***P < 0.001 vs. Mod.

Following the establishment of the model, the kidney index in the Mod group was significantly reduced (*P* < 0.001), but it recovered after drug administration (Figure 3A). Measurements of Scr and BUN levels revealed that, compared to the Con group, the Mod group exhibited significantly elevated Scr and BUN levels (*P* < 0.001), indicating impaired renal function and reduced excretory capacity (Figures 3B, C). In contrast, all three positive control drug groups and the three GP treatment groups significantly downregulated Scr and BUN levels, with the degree of reduction increasing with higher GP doses. Notably, the GP-H group (*P* < 0.001) demonstrated superior efficacy in reducing Scr levels compared to the three positive control drug groups, while both the GP-H (*P* < 0.001) and GP-M (*P* < 0.001) groups showed greater efficacy in lowering BUN levels than the positive control drugs. These results suggest that GP may exert a protective effect against renal injury in rats.

Histopathological analysis (Figure 3D) revealed that HUA rats exhibited severe renal damage, characterized by renal tubular atrophy, loosely stained tubular epithelial cells, interstitial connective tissue hyperplasia, tubular dilation, lymphocyte infiltration, and extensive UA deposition. Treatment with GP and positive control drugs significantly ameliorated these renal injuries. Notably, the GP-H group demonstrated a more pronounced effect in alleviating renal damage, consistent with the results of Scr and BUN level measurements.

### 3.4 GP exerts joint protection by alleviating inflammation in HUA rats

**Figure 4:**
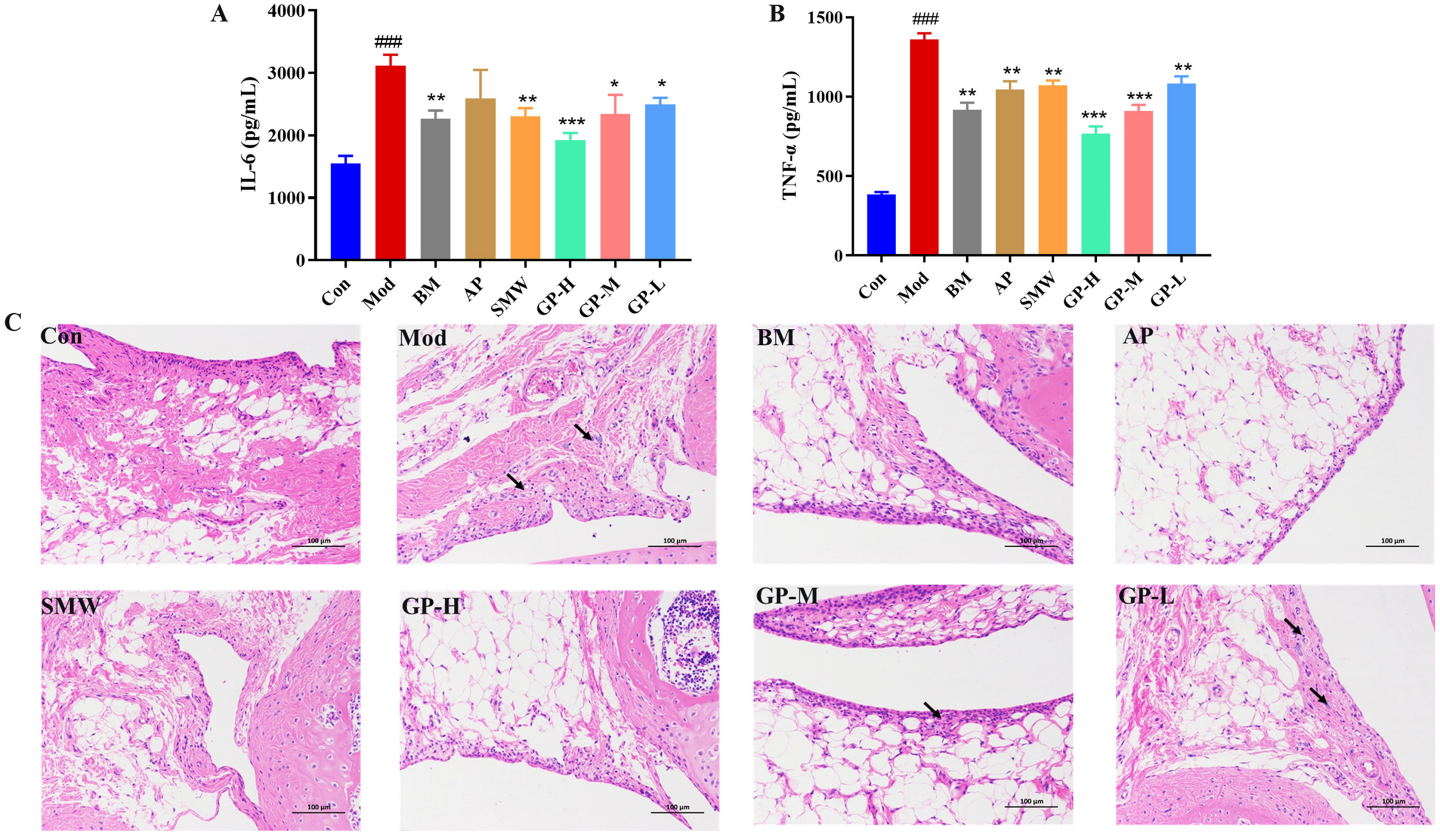
GP exhibited Anti-inflammatory effect and joint protection. **(A)** IL-6. **(B)** TNF-α. **(C)** Ameliorative effect of GP on joint tissue pathological alterations in HUA rats (HE staining images of joint sections at magnification 200×). Black arrows: punctate infiltration of lymphocytes and mast cells. Data are presented as mean ± S.E.M (*n* = 8). ^###^*P* < 0.001 vs. Con; *P < 0.05, **P < 0.01, ***P < 0.001 vs. Mod.

Excessive UA in the body is associated with increased inflammation. To evaluate this association, we measured the levels of inflammatory cytokines IL-6 and TNF-α in serum. As shown in Figure 4A, compared to the Con group, the Mod group exhibited a significant increase in serum IL-6 levels (*P* < 0.001). Treatment with benzbromarone and Simiao pill significantly reduced serum IL-6 levels (*P* < 0.01), while allopurinol showed a decreasing trend in IL-6 levels, though the difference was not statistically significant. All three GP treatment groups also demonstrated significant reductions in IL-6 levels, with the GP-H group showing superior efficacy compared to the BM and SMW groups. As illustrated in Figure 4B, the Mod group displayed significantly elevated serum TNF-α levels compared to the Con group (*P* < 0.01). Both the positive control drug groups and the GP treatment groups significantly reduced TNF-α levels, with the GP-H and GP-M groups exhibiting more pronounced effects. These findings indicate that GP can reduce serum IL-6 and TNF-α levels in a dose-dependent manner. Moreover, GP demonstrated superior protective effects compared to the positive control drugs, highlighting its potential in alleviating hyperuricemia-related inflammation.

To further assess the impact of GP on joint morphology in HUA rats, we analyzed hematoxylin and eosin (H&E)-stained sections of joint tissues. Figure 4C presents the H&E staining results, revealing focal infiltration of lymphocytes and mast cells in the Mod group, indicative of inflammatory responses in joint tissues. In contrast, the positive control drug groups showed significant improvements, and the GP-H group exhibited a marked reduction in pathological joint inflammation.

### 3.5 GP alleviates serum metabolic dysregulation in HUA rats

#### 3.5.1 Metabolomics analysis

To investigate the characteristics of serum metabolites associated with HUA and evaluate the protective effects of GP, we performed LC-MS analysis (Supplementary Figure S2). To ensure the stability of the instrument and methodology, QC samples were analyzed after every 8 experimental samples. As shown in Supplementary Figure S3, the principal component analysis (PCA) plot generated in unsupervised mode demonstrated good clustering of QC samples.

**Figure 5:**
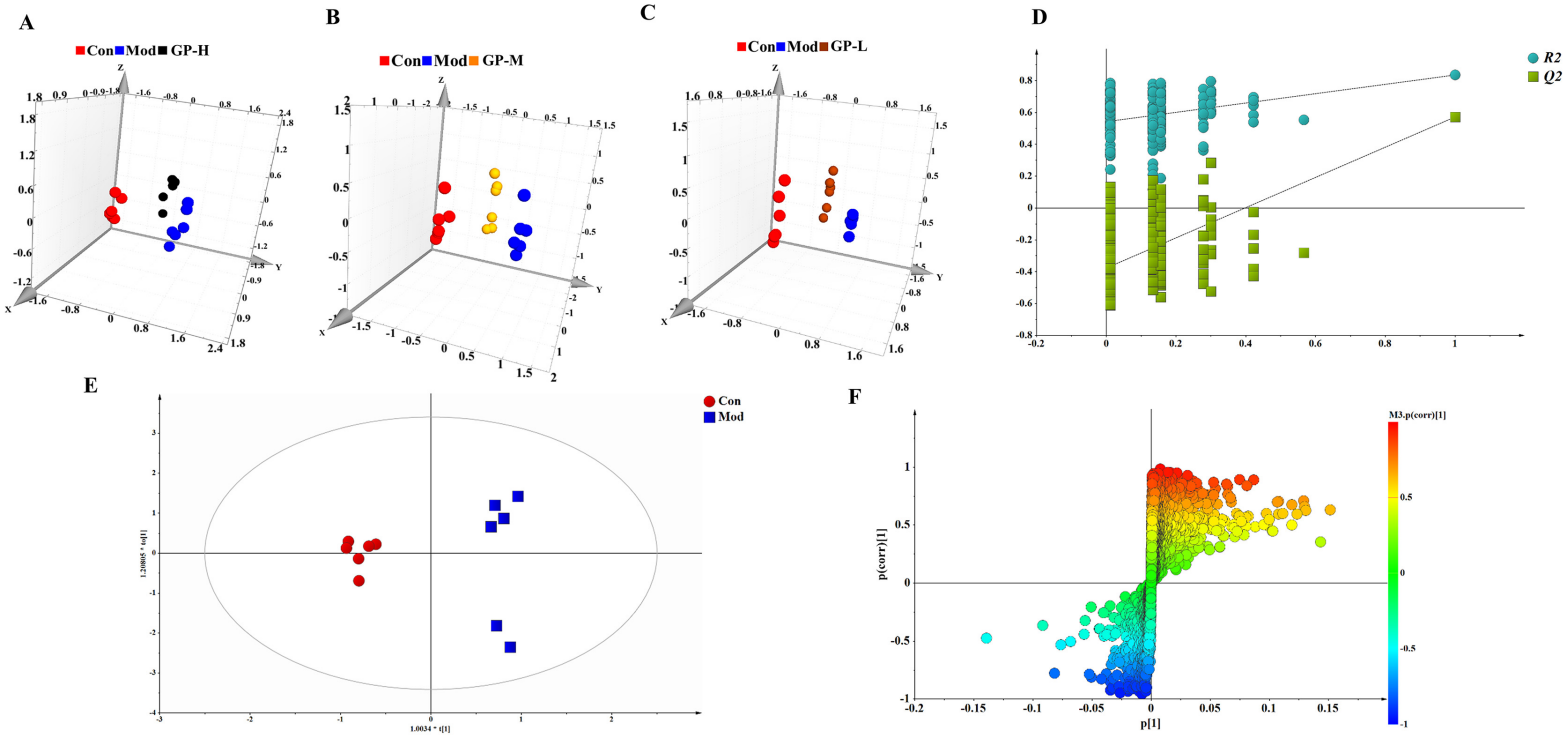
Serum metabolomics analysis (*n* = 6). PLS-DA scores plot of serum samples collected from rats. **(A)** GP-H. **(B)** GP-M. **(C)** GP-L. **(D)** Statistical validation of the corresponding PLS-DA model by permutation analysis (permutation No.: 200). **(E)** OPLS-DA score plots, and **(F)** corresponding S-plot. In **(D)**, *R*^2^ is the explained variance, and *Q*^2^ is the predictive ability of the model.

Partial least squares discriminant analysis (PLS-DA) was conducted to compare the Con, Mod, and GP groups (Figures 5A–C). The results revealed clear separation among the three groups, suggesting significant differences in endogenous serum metabolites. To further explore the metabolic differences between the Con and Mod groups and identify potential biomarkers related to the therapeutic effects of GP, orthogonal partial least squares discriminant analysis (OPLS-DA) and independent sample *t*-tests were performed. Model validation results (Figure 5D) showed that the slopes of the two regression lines were steep, and the *R*^2^ and *Q*^2^ values from any random permutation on the left were smaller than those on the right, indicating that the predictive ability of the original model was superior to any random permutation of the y-variable. This confirmed the validity of the model, demonstrating statistically significant differences between the two groups. Moreover, the OPLS-DA score plot displayed distinct clustering of the Con and Mod groups (Figure 5E), suggesting that the administration of PO and adenine disrupted metabolic pathways in normal rats.

**Figure 6:**
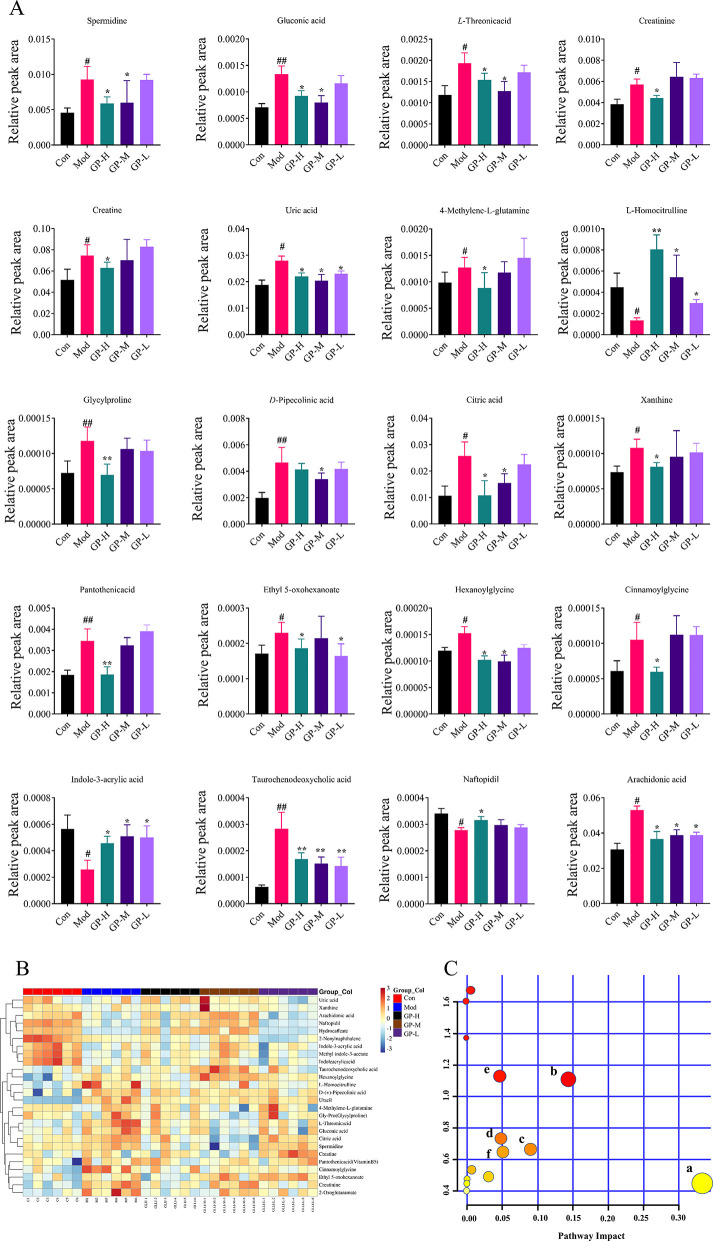
GP modulates serum differential metabolites and their metabolic pathways in HUA rats. **(A)** Comparison of relative peak areas of the potential biomarkers in serum metabolic. **(B)** Heat map of serum differential metabolites in HUA rats between groups. **(C)** Pathway analysis of differential metabolites in serum samples: (a) arachidonic acid metabolism, (b) pyrimidine metabolism, (c) citric acid cycle, (d) purine metabolism, (e) arginine and proline metabolism, (f) pentose phosphate pathway. ^#^*P* < 0.05, ^##^*P* < 0.01 vs. Con; *P < 0.05, **P < 0.01, vs. Mod.

Differential metabolites between the Con and Mod groups were screened based on VIP > 1, *P* < 0.05, and S-plot analysis (Figure 5F). As shown in Table 2, a total of 26 differential metabolites were identified. The recovery of these 26 metabolites after GP treatment was analyzed (Figures 6A, B). The results revealed that 19 metabolites were significantly restored in the GP-H group, including spermidine, gluconic acid, L-threonic acid, creatinine, creatine, 4-methylene-L-glutamine, L-homocitrulline, glycylproline, citric acid, UA, xanthine, pantothenic acid, ethyl 5-oxohexanoate, hexanoylglycine, cinnamoylglycine, indole-3-acrylic acid, taurochenodeoxycholic acid, naftopidil, and arachidonic acid. In the GP-M group, 11 metabolites showed significant recovery, while 7 metabolites were significantly restored in the GP-L group. These findings indicate that GP can ameliorate serum metabolic disturbances in HUA rats in a dose-dependent manner.

**Table 2:** The effect of GP on different metabolites related to HUA in serum of rats.

**No**.	**Metabolites**	**Formula**	**Mass (m/z)**	**RT (min)**	**Ion**	**VIP**	**Trend**			
							**M/C**	**GP-H/M**	**GP-M/M**	**GP-L/M**
1	Spermidine	C_7_H_19_ N_3_	145.1579	1.01	[M+H]^+^	1.11	↑#	↓*	↓*	↓
2	Gluconic acid	C_6_H_12_O_7_	196.0584	1.27	[M-H]^−^	1.01	↑##	↓*	↓*	↓
3	*L*-Threonic acid	C_4_H_8_O_5_	136.0373	1.27	[M-H]^−^	1.28	↑#	↓*	↓*	↓
4	Creatinine	C_4_H_7_N_3_O	113.0590	1.30	[M+H]^+^	1.44	↑#	↓*	↓	↓
5	Creatine	C_4_H_9_N_3_O_2_	131.0696	1.33	[M+H]^+^	1.10	↑#	↓*	↓	↓*
6	Uracil	C_4_H_4_N_2_O_2_	112.0272	1.35	[M-H]^−^	1.32	↑#	↓	↓	↓
7	4-Methylene-L-glutamine	C_6_H_10_N_2_O_3_	158.0691	1.37	[M+H]^+^	1.56	↑#	↓*	↓	↓
8	*L*-Homocitrulline	C_7_H_15_N_3_O_3_	189.1112	1.37	[M+H]^+^	1.20	↓#	↑**	↑*	↑*
9	Glycylproline	C_7_H_12_N_2_O_3_	172.0846	1.44	[M+H]^+^	1.22	↑##	↓**	↓	↓
10	2-Oxoglutaramate	C_5_H_7_NO_4_	145.0376	1.54	[M-H]^−^	1.16	↑#	↑	↑	↑
11	*D*-Pipecolinic acid	C_6_H_11_NO_2_	129.0790	1.58	[M+H]^+^	1.26	↑##	↓	↓*	↓
12	Citric acid	C_6_H_8_O_7_	192.0271	1.59	[M-H]^−^	1.30	↑#	↓*	↓*	↓
13	Uric acid	C_5_H_4_N_4_O_3_	168.0284	1.59	[M-H]^−^	1.70	↑#	↓*	↓*	↓*
14	Xanthine	C_5_H_4_N_4_O_2_	152.0335	1.60	[M-H]^−^	1.45	↑#	↓*	↓	↓
15	Pantothenic acid	C_9_H_17_NO_5_	219.1106	1.65	[M+Na]^+^	1.24	↑##	↓**	↓	↓
16	Hydrocaffeate	C_9_H_10_O_4_	182.0581	5.69	[M-H]^−^	1.26	↓#	↓	↑	↓
17	Ethyl 5-oxohexanoate	C_8_H_15_NO_3_	158.1053	8.77	[M-H]^−^	1.21	↑#	↓*	↓	↓*
18	Hexanoylglycine	C_8_H_15_NO_3_	173.1052	6.81	[M-H]^−^	1.14	↑#	↓*	↓*	↓
19	Cinnamoylglycine	C_11_H_11_NO_3_	205.0739	7.13	[M-H]^−^	1.12	↑#	↓*	↓	↓
20	Indole-3-acrylic acid	C_11_H_9_NO_2_	187.0633	7.89	[M+H]^+^	1.39	↓#	↑*	↑*	↑*
21	Methyl indole-3-acetate	C_11_H_11_NO_2_	189.0789	8.36	[M+H]^+^	1.40	↓#	↑	↑	↑
22	Taurochenodeoxycholic acid	C_26_H_45_NO_6_S	499.2976	9.08	[M-H]^−^	1.73	↑##	↓**	↓**	↓**
23	Indoleacrylic acid	C_11_H_9_NO_2_	187.0633	9.20	[M+H]^+^	1.24	↓#	↑	↑	↑
24	Naftopidil	C_24_H_28_N_2_O_3_	392.2116	17.16	[M+H]^+^	1.13	↓#	↑*	↑	↑
25	2-Nonylnaphthalene	C_19_H_26_	254.2034	25.99	[M+H]^+^	1.26	↓#	↑	↑	↓
26	Arachidonic acid	C_20_H_32_O_2_	304.2404	26.33	[M+H]^+^	1.67	↑#	↓*	↓*	↓*

↑ and ↓ represent higher and lower level;

# *P* < 0.05,

## *P* < 0.01 vs. Con;

* *P* < 0.05,

** *P* < 0.01 vs. Mod.

#### 3.5.2 KEGG pathway analysis

Next, we correlated and analyzed the differential metabolites using the KEGG database, along with relevant domestic and international literature. The Metascape enrichment analyses identified specific changes in differential metabolites, highlighting pathways such as arachidonic acid metabolism, pyrimidine metabolism, the citric acid cycle, arginine and proline metabolism, purine metabolism, and the pentose phosphate pathway, which may be associated with HUA and the therapeutic effects of GP (Figure 6C). Notably, the metabolites that showed significant alterations were emphasized in the purine metabolism pathway map. Our results suggest that the observed trends in metabolites align with the effects of inhibiting ADA and XOD, indicating that GP reduces SUA levels by inhibiting the activities of both enzymes. HUA is characterized by various disruptions in metabolic pathways, and after administering GP, we observed a pattern of recovery, suggesting that GP can effectively modulate the metabolic disturbances associated with HUA.

### 3.6 Network pharmacology analysis and molecular docking

#### 3.6.1 Target prediction and protein-protein interaction (PPI) analysis

**Figure 7:**
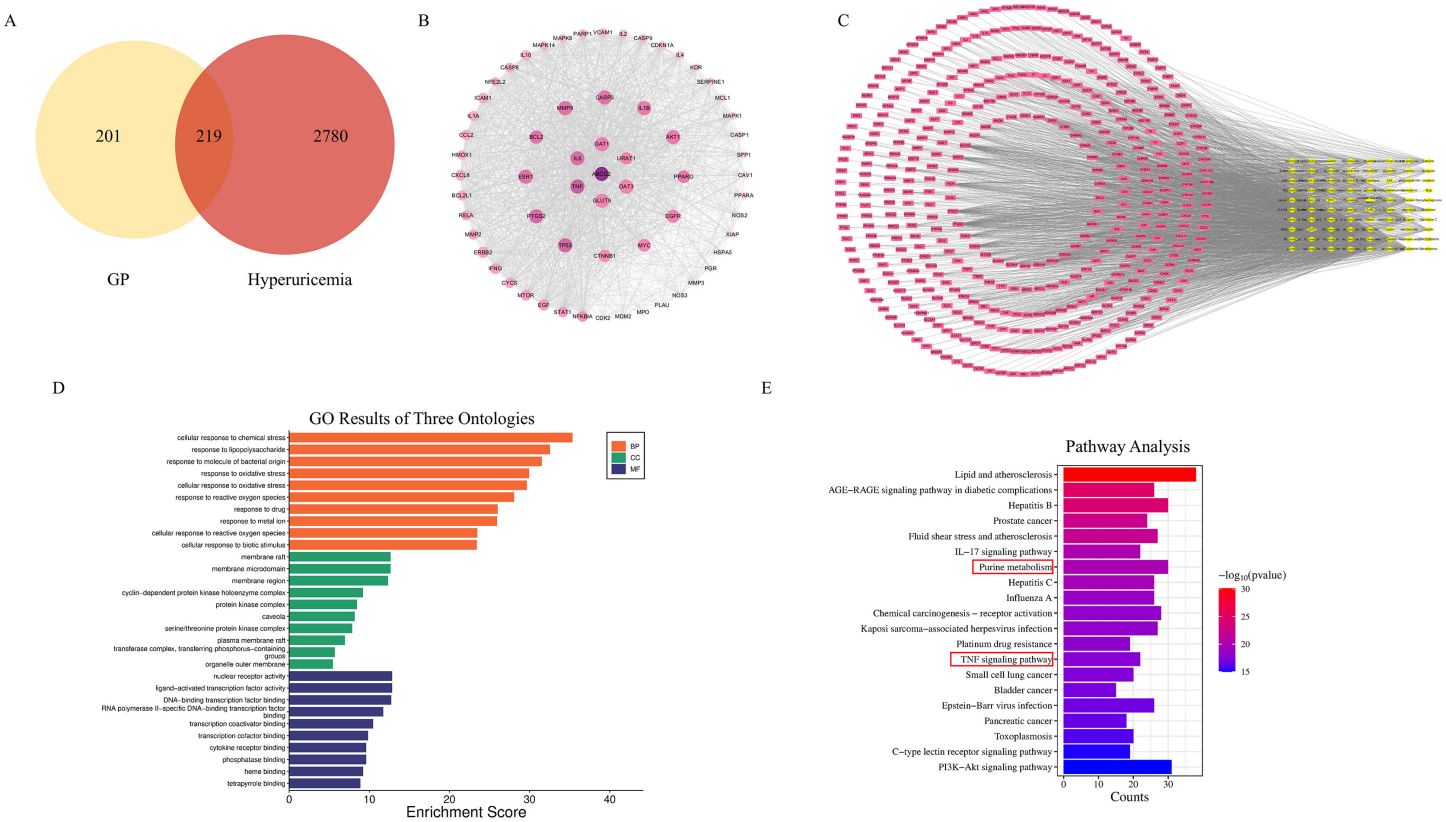
Network pharmacology predicts the effect of GP on HUA. **(A)** Venn diagram of GP components and HUA targets. **(B)** PPI network of drug-disease interactions. **(C)** Compound-target-pathway network. **(D)** GO enrichment analysis. **(E)** KEGG enrichment analysis.

To identify potential targets of GP for the treatment of HUA, we conducted a network pharmacology analysis. A total of 420 targets associated with GP were collected from the TCMSP, SwissTargetPrediction, HERB, and ETCM databases. Additionally, 2,999 disease-related gene targets associated with HUA were obtained from the GeneCards, OMIM, TTD, and DisGeNET databases. As shown in Figure 7A, 219 overlapping gene targets were identified as potential therapeutic targets of GP for HUA. The 219 targets were imported into Cytoscape to construct a PPI network (Figure 7B). PPI network analysis revealed that GP may exert inhibitory effects on HUA by modulating multiple targets (Figure 7C). Overall, the network demonstrated high levels of interaction among key targets, including ABCG2, TNF-α, IL-6, OAT1, GLUT9, OAT3, and URAT1 (Figure 7B).

#### 3.6.2 Preliminary exploration of GP's mechanism of action on HUA

Based on the above findings, to further explore the pharmacodynamic mechanisms of GP's active components, we performed GO and KEGG functional enrichment analyses using the Metascape platform. The top 20 enriched pathways were identified (Figures 7D, E). Notably, these genes were significantly enriched in pathways related to lipid and atherosclerosis, purine metabolism, and TNF signaling, which aligned with the results from metabolomics analysis. Finally, the regulatory network of GP for HUA treatment suggested that GP alleviates HUA through a multi-component, multi-target, and multi-pathway mechanism.

#### 3.6.3 Molecular docking

**Figure 8:**
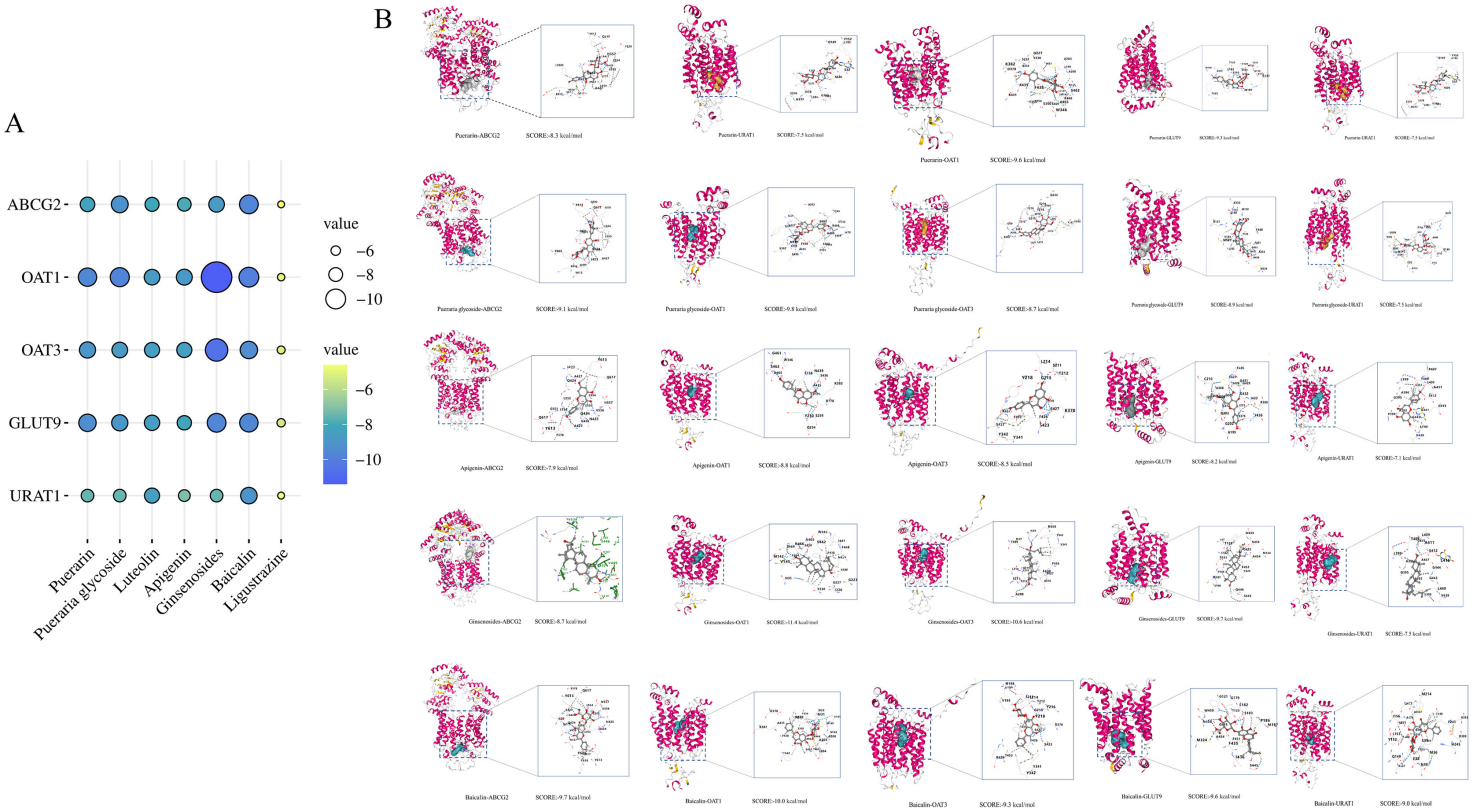
GP core components are interconnected with molecules of core targets. **(A)** Total score bubble map. **(B)** Mapping of target-active ingredient docking patterns.

Molecular docking is a computational technique used to predict the binding modes and affinities between small molecule ligands and proteins, with results expressed as binding energy. Lower binding energy indicates stronger interactions. To explore the key signaling molecules of GP in HUA and investigate the interactions between active components and core targets, molecular docking was performed using 7 selected active components and 5 core targets. As shown in Figure 8A, the binding energies of most ligand-receptor pairs were below −5.0 kcal/mol, with ginsenoside exhibiting the strongest binding affinity (−11.4 kcal/mol) for the OAT1 target. The results demonstrated that the core components of GP, particularly puerarin, daidzin, luteolin, apigenin, ginsenoside, and baicalin, exhibited high affinity for the core targets. Notably, ginsenoside showed the strongest binding affinity and highest activity (Figure 8B).

### 3.7 GP regulates UA levels by modulating renal UA transporters in HUA rats

**Figure 9:**
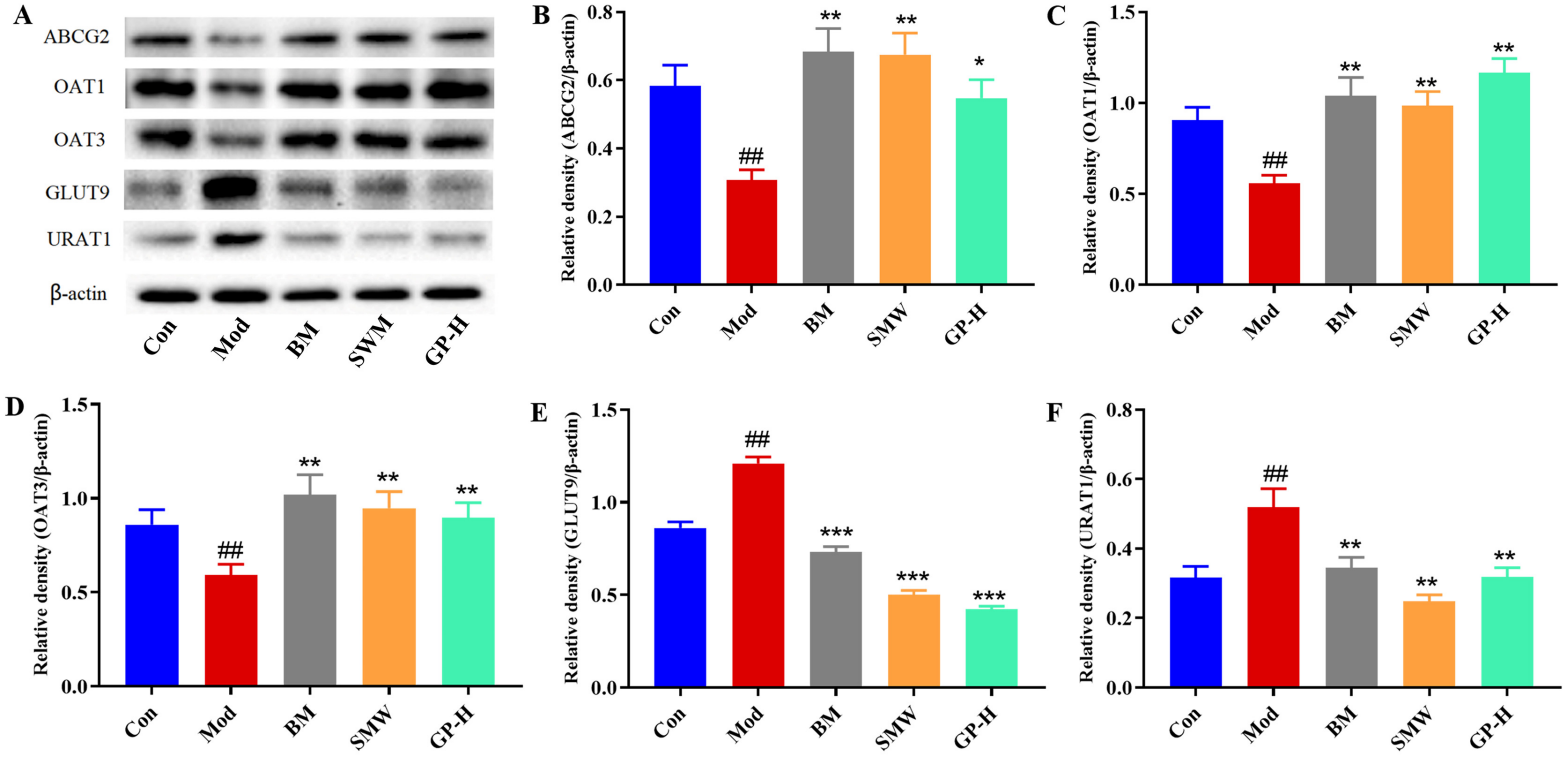
GP promoted UA excretion through modulation of renal UA transporters in HUA rats. **(A)** Expression of ABCG2, OAT1, OAT3, and GLUT9, URAT1 in rat kidney. **(B–F)** The western blot analysis of ABCG2, OAT1, OAT3, and GLUT9, URAT1 relative protein expression levels. Data are presented as mean ± S.E.M (*n* = 3). ^##^*P* < 0.01 vs. Con; *P < 0.05, **P < 0.01, ***P < 0.001 vs. Mod.

Uric acid transporters are a class of proteins responsible for the transport of UA in the body, playing a crucial role in maintaining UA homeostasis. These transporters primarily include UA secretion transporters, such as ABCG2, OAT1, and OAT3, as well as UA reabsorption transporters, such as GLUT9 and URAT1. In this study, we evaluated the relative expression levels of renal UA transporters in HUA rats before and after treatment (Figure 9A). The results showed that, compared to the Con group, the expression levels of UA secretion transporters ABCG2, OAT1, and OAT3 were significantly reduced in the Mod group (*P* < 0.01) (Figures 9B–D). This reduction indicates impaired UA excretion, which may adversely affect overall metabolic health. In contrast, GP treatment significantly restored the expression of these transporters: ABCG2 (*P* < 0.05), OAT1 (*P* < 0.01), and OAT3 (*P* < 0.01). These findings suggest that GP effectively promotes UA excretion, bringing transporter expression levels closer to those observed with positive control drugs BM and SMW. Furthermore, compared to the Con group, the expression levels of UA reabsorption transporters GLUT9 and URAT1 were significantly elevated in the Mod group (*P* < 0.01) (Figures 9E, F). However, in the BM group, SMW group, and GP-H group, the expression levels of GLUT9 and URAT1 were significantly reduced (*P* < 0.01). This indicates that GP has a similar effect to positive control drugs in mitigating abnormal UA reabsorption and maintaining UA homeostasis. These findings are supportive and should be confirmed in future studies with larger sample sizes.

## 4 Discussion

**Figure 10:**
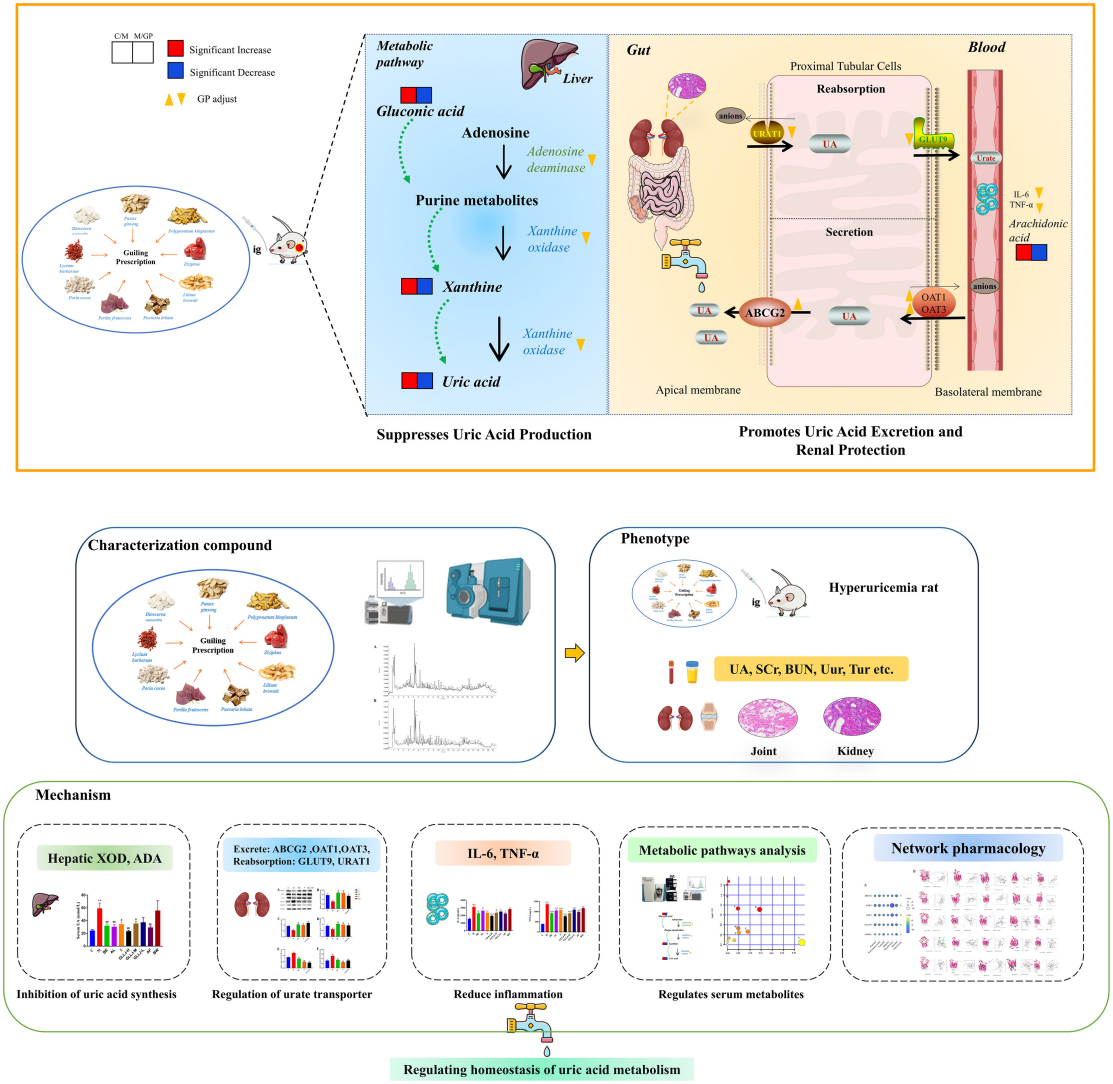
GP alleviates HUA by promoting UA excretion, reducing purine synthesis and regulating metabolic pathway disorders.

This study evaluated the therapeutic efficacy of GP in a HUA rat model induced by PO and adenine. The success of the model was confirmed by measuring SUA levels and examining tissue morphology using hematoxylin-eosin staining of the kidneys and joints. To assess the impact of GP on renal function, Scr and urea nitrogen levels were measured, and inflammatory factors were analyzed to determine the inflammatory status of the rats. This investigation aimed to elucidate the mechanisms by which GP alleviates HUA, focusing on its effects on UA transporters involved in UA metabolism. Additionally, metabolomics and network pharmacology approaches were employed to examine GP's influence on metabolic profiles. The results demonstrated that GP significantly modulates these profiles, particularly in relation to purine metabolism. Both metabolomic and network pharmacology analyses further revealed that GP reduces inflammatory responses and promotes a more favorable metabolic environment. In conclusion, these findings suggest that GP effectively delays the progression of HUA, underscoring its therapeutic potential (Figure 10). A key strength of this study is the comprehensive comparison of GP with both Western and Chinese medicines, which enhances our understanding of its clinical value and applications in HUA treatment.

HUA is primarily defined by an increase in UA levels in the bloodstream (27). The liver plays a central role in purine metabolism, with two key enzymes—ADA and XOD—involved in the synthesis of UA. XOD is responsible for converting hypoxanthine into xanthine and further oxidizing it to produce UA. Concurrently, ADA aids in transforming adenine nucleotides into hypoxanthine, thereby indirectly impacting UA formation (28). In our investigation, GP demonstrated a significant reduction in SUA levels in rats with HUA, confirming its effectiveness in lowering UA concentrations. Moreover, GP showed a notable inhibitory effect on the activities of both XOD and ADA, suggesting that it may help decrease the internal synthesis of UA. This indicates that GP could potentially downregulate the expression of XOD and ADA in the liver, facilitating liver repair, which subsequently contributes to the reduction of UA levels and the alleviation of HUA. While preliminary analysis of hepatic enzymes showed no signs of acute hepatotoxicity, a comprehensive evaluation of the long-term safety profile of GP still requires future systematic chronic toxicity studies conducted in accordance with standard toxicological guidelines.

Approximately 90% of individuals diagnosed with HUA have elevated UA levels, primarily due to impaired kidney function (29). Two critical proteins, URAT1 and GLUT9, are involved in the reabsorption of urate from the kidney lumen into the proximal tubule cells (30). Other transporters, including ABCG2, OAT1, and OAT3, also play significant roles in urate secretion. Previous research has identified these proteins as promising therapeutic targets for managing HUA (31).

This investigation focuses on how GP enhances the excretion of UA through renal pathways. Notable findings include a substantial increase in URAT1 and GLUT9 protein levels, alongside a decrease in ABCG2, OAT1, and OAT3 expression in HUA rat models. Following GP administration, the expression of these transporters was notably altered. These results indicate that GP affects the activity of renal UA transporters, thereby facilitating increased UA elimination and presenting a potential approach for addressing HUA. Notably, GP treatment significantly increased fecal uric acid excretion, suggesting a potential role for intestinal elimination. While this study did not investigate the gut microbiome, emerging evidence highlights its critical function in uric acid homeostasis via bacterial degradation, modulation of intestinal barrier integrity, and systemic inflammation (32). The increase in fecal urate following GP administration may involve shifts in microbial communities, such as an enrichment of uricase-producing bacteria or changes in microbial metabolites (33). This represents a compelling mechanistic avenue for future research. A dedicated study combining 16S rRNA sequencing, metagenomics, and metabolomics of fecal samples is warranted to characterize GP-induced microbial alterations and establish causal links to its anti-hyperuricemic effects (34).

Creatinine and BUN are important markers of kidney function (35). Elevated levels of both substances often indicate impaired renal function and reduced clearance (36). Additionally, inflammation plays a critical role in HUA and contributes to renal injury associated with this condition. The presence of proinflammatory cytokines IL-6 and TNF-α in the kidney serves as indicators of renal and joint damage (37). In this study, the administration of PO and adenine resulted in significant increases in Scr, BUN, IL-6, and TNF-α levels. Observations also included renal interstitial edema, enlarged renal tubules, urate deposits in the kidneys, and lymphocyte and mast cell infiltration in the joints. Notably, GP effectively lowered elevated levels of creatinine and BUN, indicating its potential therapeutic effects on nephropathy associated with HUA. Histopathological examinations, including H&E staining, further confirmed GP's protective effects, showing reduced histopathological lesions following treatment. Overall, GP demonstrated greater efficacy than the positive control drug in lowering these indicators in HUA rats. These findings suggest that GP may protect the kidneys and joints through its anti-inflammatory properties. Future studies incorporating direct measurement of inflammatory cytokines in synovial fluid or joint tissue homogenates are warranted to further validate the local anti-inflammatory mechanism. Moreover, the pathogenesis of hyperuricemia involves key signaling pathways—including TLR4/MyD88/NF-κB, TGF-β/Smad, and JAK/STAT—which contribute to oxidative stress and inflammatory responses (38). Notably, active components in GP, such as puerarin, have been shown to inhibit NF-κB and modulate the TGF-β/Smad pathway (39). Therefore, further investigation into these multi-mechanistic actions is essential to fully elucidate the multi-target therapeutic advantage of GP.

The investigation into variations in serum metabolites seeks to recognize early and distinct metabolic indicators of diseases, all while assessing how drug interventions impact these conditions (40). Utilizing serum metabolomics to investigate the mechanism by which GP alleviates HUA can effectively address the limitations of traditional pharmacological experiments and facilitate a comprehensive understanding of GP's effects from a holistic perspective. This research identified six metabolic pathways related to HUA in the serum: the metabolism of arachidonic acid, pyrimidine metabolism, the citric acid cycle, the metabolism of arginine and proline, purine metabolism, and the pentose phosphate pathway.

Arachidonic acid is one of the most widely distributed unsaturated fatty acids with important biological activity in the body (41). When the body detects inflammatory substances, arachidonic acid is released into the cell fluid and converted into cyclooxygenase, lipoxygenase, and cytochrome P450, and then induces an inflammatory response. The arachidonic acid metabolic network is the principal pathway responsible for producing inflammatory mediators and initiating inflammation, playing a crucial role in its development (42). Research has shown that sustained high levels of UA may lead to the accumulation of urate in the kidneys, joints, cartilage, and various other tissues, consequently initiating a series of inflammatory responses (43). This research indicates that after the oral intake of GP, there was a notable reduction in arachidonic acid levels. This suggests a potential link between the anti-hyperuricemia effects of GP and its anti-inflammatory characteristics, which is consistent with the results obtained from our pharmacological experiments. Notably, this anti-inflammatory effect can be attributed to the “heat-clearing and blood-activating” herbs in GP, such as Pueraria lobata (Gegen) and Perilla frutescens (Zisuye). Compounds like puerarin (from Gegen) and apigenin/flavonoids (from Zisuye) have documented inhibitory effects on arachidonic acid metabolism and downstream inflammatory signaling, providing a phytochemical basis for this metabolic correction (44, 45).

The perturbation of citric acid, a key TCA cycle intermediate, indicates impaired mitochondrial energy metabolism in HUA, likely contributing to renal tubular dysfunction (46). GP's restoration of citrate levels suggests improved cellular bioenergetics and renal tubular integrity. This action aligns with the formula's core function of “tonifying the spleen and kidneys.” Herbs such as *Dioscorea opposita* (Shanyao), *Panax ginseng* (Renshen), and *Poria cocos* (Fuling) are traditionally used to strengthen spleen qi and kidney function. Modern research indicates that polysaccharides and saponins from these herbs can enhance mitochondrial function and cellular resilience, thereby supporting the metabolic homeostasis reflected by normalized TCA cycle intermediates (47–49). The increase in spermidine in the HUA model may represent a compensatory cellular stress response, as polyamines are involved in regulating autophagy, oxidative stress, and cell survival (50). Its normalization by GP suggests a mitigation of this stress state and a potential promotion of protective autophagy, a process crucial for combating metabolic and inflammatory tissue damage. This regulatory effect may be linked to the antioxidant and nephroprotective properties of multiple GP components, including *Lycium barbarum* (Gouqizi) and *Polygonatum kingianum* (Huangjing), which are rich in bioactive compounds known to modulate stress-response pathways (51, 52).

Deoxyribonucleic acid and ribonucleic acid serve as the fundamental genetic materials for all forms of life. These biomolecules are ubiquitous and play a crucial role in the hereditary processes of living organisms. Uracil undergoes a series of deamination, reduction, and hydrolysis in the body to be converted into β-amino acids, subsequently affecting the synthesis and metabolism of amino acids (53). In this study, an increase in uracil levels was detected in the Mod group, indicating a disorder in pyrimidine metabolism in HUA rats, with a significant trend toward normalization following treatment. Additionally, during purine degradation, xanthine is synthesized, which is ultimately converted into UA under the catalysis of XOD. The Mod group showed a marked rise in serum xanthine levels, which corresponded with an increase in UA concentrations. This phenomenon may result from the extensive transformation and accumulation of adenine, simulating a high-purine diet in humans. After administration of GP, both xanthine and UA levels decreased, suggesting that GP may exert its anti-hyperuricemia effects by inhibiting the conversion of xanthine to UA.

The pentose phosphate pathway provides reducing agents for various biological reactions, and its intermediate products serve as essential raw materials for substance synthesis in the body. Among them, 5-phosphate ribose is an important intermediate product in the pentose phosphate pathway, and is an important raw material for the synthesis of purine nucleotides (54). In this study, the content of gluconic acid was found to be significantly elevated in the HUA Mod group. A notable trend toward normalization was observed following treatment with GP, indicating that GP effectively improves the disruption of the pentose phosphate pathway caused by HUA. Additionally, the significant increase in gluconic acid may directly influence the synthesis of ribose-5-phosphate, subsequently impacting purine metabolism in the body. Thus, GP's modulation of this metabolite may represent an upstream regulatory strategy to limit purine nucleotide precursor availability, complementing its direct inhibition of XOD (55). The alterations in uracil (pyrimidine) and xanthine (purine) highlight a broad dysregulation of nucleotide metabolism in HUA. GP's corrective effect on these pathways underscores its systemic restorative action. The reduction in xanthine directly visualizes the inhibition of XOD activity, a key urate-lowering mechanism shared by several GP herbs like *Pueraria lobata* and *Lycium barbarum*. While the endpoint metabolomics data provide a snapshot of the overall metabolic restoration induced by GP, this single time-point design does not capture the dynamic reprogramming of metabolic pathways. Future studies employing serial metabolomic profiling could delineate the temporal sequence of GP's effects—for instance, clarifying whether anti-inflammatory modulation precedes purine metabolism normalization. Extending this with integrated transcriptomic or proteomic analyses would further map the gene/protein-metabolite networks underlying GP's multi-target pharmacology, providing a systems-level understanding of its mechanism (56).

In summary, GP demonstrated efficacy comparable or superior to positive controls (allopurinol, benzbromarone, and Simiao pill) in lowering serum urate, improving renal function, and suppressing systemic inflammation. Its multicomponent nature enables integrated multi-target effects—simultaneously inhibiting uric acid synthesis and promoting renal and intestinal excretion—while showing a favorable preliminary hepatic safety profile. Unlike conventional synthetic drugs constrained by target-specific action and side effects, GP functions as a medicinal food homologous formula, aligning holistic system modulation with food-based preventive healthcare. This positions GP not only as a therapeutic agent but also as a sustainable, low-risk option for the long-term dietary management of hyperuricemia and related metabolic comorbidities, reflecting a convergence of traditional wisdom and contemporary nutritional science.

It is important to acknowledge two interrelated limitations of this systems-level study. First, while our integrated analyses predict multi-target actions, evaluating only the complete GP formula precludes definitive attribution of the observed effects to specific herbal components or their synergies. Although molecular docking suggests strong binding affinities for key compounds such as ginsenosides and puerarin, these computational predictions require experimental validation through techniques like surface plasmon resonance (SPR) to quantify binding dynamics and confirm individual contributions. Second, as a result, direct experimental links between specific components and critical pathway targets remain to be established, such as NF-κB.

Future studies should therefore adopt a multi-tiered strategy combining reductionist and phenotypic approaches. This would involve comparative pharmacodynamic profiling of individual herbs and their bioactive compounds coupled with *in vitro* target-validation assays to delineate composition-activity relationships. In parallel, phenotypic models—such as those assessing inhibition of monosodium urate crystal deposition—should be implemented to provide functional validation of the formula's integrated effects. These directions will be essential to further elucidate and optimize GP's therapeutic profile.

## 5 Conclusions

This study has demonstrated the protective effects of GP in HUA rats, along with the metabolic pathways involved. Our findings indicate that GP alleviates HUA by inhibiting purine metabolism enzymes, enhancing UA excretion, regulating UA transporter expression in the kidneys, and mitigating renal and joint damage, as well as the inflammatory responses associated with elevated SUA levels. Additionally, GP appears to modulate metabolic pathways, including arachidonic acid metabolism, pyrimidine metabolism, purine metabolism, and the citric acid cycle. Overall, these studies suggest that, from the perspective of medicinal and edible homology and multi-target therapeutic effects, GP holds the potential to be developed into an innovative nutritional supplement with dual functions of treatment and metabolic regulation for patients with hyperuricemia and gout.

## Data Availability

The raw data supporting the conclusions of this article will be made available by the authors, without undue reservation.

## References

[1] Dalbeth N Gosling AL Gaffo A Abhishek A. Gout. Lancet. (2021) 397:1843–55. doi: 10.1016/S0140-6736(21)00569-9 33798500

[2] Liu J Tao L Zhao Z Mu Y Zou D Zhang J . Two-year changes in hyperuricemia and risk of diabetes: a five-year prospective cohort study. J Diabetes Res. (2018) 2018:6905720. doi: 10.1155/2018/6905720 30693289 PMC6332976

[3] Zhang Y Li Y Li C Zhao Y Xu L Ma S . Paeonia × suffruticosa Andrews leaf extract and its main component apigenin 7-O-glucoside ameliorate hyperuricemia by inhibiting xanthine oxidase activity and regulating renal urate transporters. Phytomedicine. (2023) 118:154957. doi: 10.1016/j.phymed.2023.154957 37478683

[4] Kuwabara M Kodama T Ae R Kanbay M Andres-Hernando A Borghi C . Update in uric acid, hypertension, and cardiovascular diseases. Hypertens Res. (2023) 46:1714–26. doi: 10.1038/s41440-023-01273-3 37072573

[5] Johnson RJ Bakris GL Borghi C Chonchol MB Feldman D Lanaspa MA . Hyperuricemia, acute and chronic kidney disease, hypertension, and cardiovascular disease: report of a scientific workshop organized by the National Kidney Foundation. Am J Kidney Dis. (2018) 71:851–65. doi: 10.1053/j.ajkd.2017.12.009 29496260 PMC7286363

[6] Ragab G Elshahaly M Bardin T. Gout: an old disease in new perspective - a review. J Adv Res. (2017) 8:495–511. doi: 10.1016/j.jare.2017.04.008 28748116 PMC5512152

[7] Gu X Hao D Xiao P. Research progress of Chinese herbal medicine compounds and their bioactivities: fruitful 2020. Chin Herb Med. (2022) 14:171–86. doi: 10.1016/j.chmed.2022.03.004 36117669 PMC9476823

[8] Ding R. Research on the Anti-Hyperuricaemia Effect of Guilinglou Wine and its Mechanism. Shanxi University (2022).

[9] Sung YY Yuk HJ Kim DS. Saengmaeksan, a traditional herbal formulation consisting of Panax ginseng, ameliorates hyperuricemia by inhibiting xanthine oxidase activity and enhancing urate excretion in rats. J Ginseng Res. (2021) 45:565–74. doi: 10.1016/j.jgr.2021.01.001 34803426 PMC8587482

[10] Huo LN Wang W Zhang CY Shi HB Liu Y Liu XH . Bioassay-guided isolation and identification of xanthine oxidase inhibitory constituents from the leaves of Perilla frutescens. Molecules. (2015) 20:17848–59. doi: 10.3390/molecules201017848 26425999 PMC6331977

[11] Yu D Wang Y Yu C Song M Zhou Q Liu S . High-throughput serum metabolomics analysis of gouty arthritis rat treated by total saponins of Rhizoma Dioscoreae Makino by UPLC-Q/TOF-MS. Biomed Chromatogr. (2020) 34:e4867. doi: 10.1002/bmc.4867 32330320

[12] Liu CM Ma JQ Sun YZ. Puerarin protects rat kidney from lead-induced apoptosis by modulating the PI3K/Akt/eNOS pathway. Toxicol Appl Pharmacol. (2012) 258:330–42. doi: 10.1016/j.taap.2011.11.015 22172631

[13] Yu X Zhang L Zhang P Zhi J Xing R He L . Lycium barbarum polysaccharides protect mice from hyperuricaemia through promoting kidney excretion of uric acid and inhibiting liver xanthine oxidase. Pharm Biol. (2020) 58:944–9. doi: 10.1080/13880209.2020.1817951 32946701 PMC7534190

[14] Mo SF Zhou F Lv YZ Hu QH Zhang DM Kong LD . Hypouricemic action of selected flavonoids in mice: structure-activity relationships. Biol Pharm Bull. (2007) 30:1551–6. doi: 10.1248/bpb.30.1551 17666819

[15] Orhan IE Deniz FSS. Natural products and extracts as xantine oxidase inhibitors - a hope for gout disease? Curr Pharm Des. (2021) 27:143–58. doi: 10.2174/1381612826666200728144605 32723252

[16] Lan Z Bi KS Chen XH. Ligustrazine attenuates elevated levels of indoxyl sulfate, kidney injury molecule-1 and clusterin in rats exposed to cadmium. Food Chem Toxicol. (2014) 63:62–8. doi: 10.1016/j.fct.2013.10.038 24200859

[17] Zhong L Lin Y Gong S Wu X Liu Y Chen J . Oxyberberrubine, a novel liver microsomes-mediated secondary metabolite of berberine, alleviates HUA nephropathy in mice. Phytomedicine. (2023) 108:15452. doi: 10.1016/j.phymed.2022.154521 36334387

[18] Li Y Zhao Z Luo J Jiang Y Li L Chen Y . Apigenin ameliorates HUA nephropathy by inhibiting URAT1 and GLUT9 and relieving renal fibrosis via the Wnt/β-catenin pathway. Phytomedicine. (2021) 87:153585. doi: 10.1016/j.phymed.2021.153585 34044255

[19] Zhang Y Su H Zhang J Kong J. The effects of ginsenosides and anserine on the up-regulation of renal aquaporins 1-4 in HUA mice. Am J Chin Med. (2019) 47:1133–47. doi: 10.1142/S0192415X19500587 31311296

[20] Liu Y Li X Chen C Ding N Ma S Yang M . Exploration of compatibility rules and discovery of active ingredients in TCM formulas by network pharmacology. Chin Herb Med. (2024) 16:572–88. doi: 10.1016/j.chmed.2023.09.008 39606260 PMC11589340

[21] Liu Y Deng W Wei F Kang X Han R Feng X . Recent advances in the application of foodborne substances in hyperuricemia. J Agric Food Chem. (2024) 72:27639–53. doi: 10.1021/acs.jafc.4c07267 39630974

[22] Yang HY Liu ML Luo P Yao XS Zhou H. Network pharmacology provides a systematic approach to understanding the treatment of ischemic heart diseases with traditional Chinese medicine. Phytomedicine. (2022) 104:154268. doi: 10.1016/j.phymed.2022.154268 35777118

[23] Alseekh S Aharoni A Brotman Y Contrepois K D'Auria J Ewald J . Mass spectrometry-based metabolomics: a guide for annotation, quantification and best reporting practices. Nat Methods. (2021) 18:747–56. doi: 10.1038/s41592-021-01197-1 34239102 PMC8592384

[24] Wei R Qin X Li Z. Comparison of the inedible parts of white and green asparagus based on metabolomics and network pharmacology. Food Funct. (2023) 14:7478–88. doi: 10.1039/D3FO01797D 37497633

[25] Liu Y Yang X Gan J Chen S Xiao ZX Cao Y . CB-Dock2: improved protein-ligand blind docking by integrating cavity detection, docking and homologous template fitting. Nucleic Acids Res. (2022) 50:W159–64. doi: 10.1093/nar/gkac394 35609983 PMC9252749

[26] Adachi SI Oyama M Kondo S Yagasaki K. Comparative effects of quercetin, luteolin, apigenin and their related polyphenols on uric acid production in cultured hepatocytes and suppression of purine bodies-induced hyperuricemia by rutin in mice. Cytotechnology. (2021) 73:343–51. doi: 10.1007/s10616-021-00452-9 34149170 PMC8167080

[27] Caliceti C Calabria D Roda A Cicero AFG. Fructose intake, serum uric acid, and cardiometabolic disorders: a critical review. Nutrients. (2017) 9:395. doi: 10.3390/nu9040395 28420204 PMC5409734

[28] Maiuolo J Oppedisano F Gratteri S Muscoli C Mollace V. Regulation of uric acid metabolism and excretion. Int J Cardiol. (2016) 213:8–14. doi: 10.1016/j.ijcard.2015.08.109 26316329

[29] Schmidt AP de Oliveira ED Fagundes AC Hansel G Pedrini RQ Valdameri A . Allopurinol attenuates postoperative pain and modulates the purinergic system in patients undergoing abdominal hysterectomy: a randomized controlled trial. J Anesth. (2021) 35:818–26. doi: 10.1007/s00540-021-02983-z 34390392

[30] Shu L Yang M Liu N Liu Y Sun H Wang S . Short hexapeptide optimized from rice-derived peptide 1 shows promising anti-hyperuricemia activities. J Agric Food Chem. (2022) 70:6679–87. doi: 10.1021/acs.jafc.2c00354 35608514

[31] So A Thorens B. Uric acid transport and disease. J Clin Invest. (2010) 120:1791–9. doi: 10.1172/JCI42344 20516647 PMC2877959

[32] Terkeltaub R Dodd D. The gut microbiome in hyperuricemia and gout. Arthritis Rheumatol. (2025) 77:955–65. doi: 10.1002/art.43118 39829115 PMC12276925

[33] Li ZX Kang KW Zheng H Li DL Xu JC Lv HQ . Puerarin-rich compound Puerariae lobatae formulas alleviate hyperuricemia in mice by enhancing renal and intestinal function through regulating gut microbiota. Phytomedicine. (2025) 146:157115. doi: 10.1016/j.phymed.2025.157115 40815946

[34] Wang C He X Rao L Liao J Qian J Yu X . Untargeted metabolomics and network pharmacology reveal the uric acid-lowering effects of alcohol extract of *Gnaphalium affine* and its extracts in hyperuricemic rats. Food Bioscience. (2025) 68:106471. doi: 10.1016/j.fbio.2025.106471

[35] Yale JF Bakris G Cariou B Yue D David-Neto E Xi . and safety of canagliflozin in subjects with type 2 diabetes and chronic kidney disease. Diabetes Obes Metab. (2013) 15:463–73. doi: 10.1111/dom.12090 23464594 PMC3654568

[36] Dai Y Chen X Yang H Yang J Hu Q Xiao X . Evidence construction of Huangkui capsule against chronic glomerulonephritis: a systematic review and network pharmacology. Phytomedicine. (2022) 102:154189. doi: 10.1016/j.phymed.2022.154189 35617887

[37] Joosten LAB Crişan TO Bjornstad P Johnson RJ. Asymptomatic hyperuricaemia: a silent activator of the innate immune system. Nat Rev Rheumatol. (2020) 16:75–86. doi: 10.1038/s41584-019-0334-3 31822862 PMC7075706

[38] Yu WC Liu JR Baranenko D Cifuentes A Ibañez E Zhang YC . The role of dietary polysaccharides in uric acid regulation: mechanisms and benefits in managing hyperuricemia. Trends Food Sci Technol. (2025) 157:104902. doi: 10.1016/j.tifs.2025.104902

[39] Xie H Chen Y Du K Wu W Feng X. Puerarin alleviates vincristine-induced neuropathic pain and neuroinflammation via inhibition of nuclear factor-κB and activation of the TGF-β/Smad pathway in rats. Int Immunopharmacol. (2020) 89(Pt B):107060. doi: 10.1016/j.intimp.2020.107060 33049496

[40] Wang LM Wang P Teka T Zhang YC Yang WZ Zhang Y . ^1^H NMR and UHPLC/Q-orbitrap-MS-based metabolomics combined with 16S rRNA gut microbiota analysis revealed the potential regulation mechanism of nuciferine in hyperuricemia rats. J Agric Food Chem. (2020) 68:14059–70. doi: 10.1021/acs.jafc.0c04985 33146009

[41] Das UN. Arachidonic acid and other unsaturated fatty acids and some of their metabolites function as endogenous antimicrobial molecules: a review. J Adv Res. (2018) 11:57–66. doi: 10.1016/j.jare.2018.01.001 30034876 PMC6052656

[42] Tunctan B Senol SP Temiz-Resitoglu M Guden DS Sahan-Firat S Falck JR . Eicosanoids derived from cytochrome P450 pathway of arachidonic acid and inflammatory shock. Prostaglandins Other Lipid Mediat. (2019) 145:106377. doi: 10.1016/j.prostaglandins.2019.106377 31586592

[43] Rock KL Kataoka H Lai JJ. Uric acid as a danger signal in gout and its comorbidities. Nat Rev Rheumatol. (2013) 9:13–23. doi: 10.1038/nrrheum.2012.143 22945591 PMC3648987

[44] Du YJ Zhao SQ Huang RY Shi YJ Meng H Dong YM . Mechanisms of Puerariae Lobatae Radix in regulating sebaceous gland secretion: insights from network pharmacology and experimental validation. Front Pharmacol. (2024) 15:1414856. doi: 10.3389/fphar.2024.1414856 39114361 PMC11303875

[45] Yoshikiyo K Yoshioka Y Narumiya Y Oe S Kawahara H Kurata K . Thermal stability and bioavailability of inclusion complexes of perilla oil with γ-cyclodextrin. Food Chem. (2019) 294:56–9. doi: 10.1016/j.foodchem.2019.04.093 31126500

[46] Rao L Dong B Chen Y Liao J Wang C Fu G . Study on the mechanism of lactic acid bacteria and their fermentation broth in alleviating hyperuricemia based on metabolomics and gut microbiota. Front Nutr. (2024) 11:1495346. doi: 10.3389/fnut.2024.1495346 39698246 PMC11652139

[47] Ren X Luo FH Chen ZH Jing J Cai YY Lu XL . Botany, traditional applications, phytochemistry, pharmacologic activities, clinical application, and quality control of Dioscoreae Rhizoma: a comprehensive review. J Ethnopharmacol. (2026) 355(Pt A):120662. doi: 10.1016/j.jep.2025.120662 41033420

[48] Xu M Chen W Liang W. Effects and mechanisms of polysaccharides from natural medicinal plants on improving aerobic exercise capacity. Front Nutr. (2025) 12:1650499. doi: 10.3389/fnut.2025.1650499 40959703 PMC12434021

[49] Chen C Liu K Wang Y Song X Gao W Wang Y . *In vitro* colonic fermentation of fermented Radix Astragali by *Poria cocos* and anti-hyperuricemia mechanism based on network pharmacology and experiment verification. Front Nutr. (2024) 11:1466702. doi: 10.3389/fnut.2024.1466702 39717393 PMC11663651

[50] Lin C Zheng Q Yu H Wu T Chen L Lin W . Uric acid-induced cardiomyocytic polyamines' insufficience: a potential mechanism mediates cardiomyocytic injury. Front Endocrinol. (2025) 16:1504614. doi: 10.3389/fendo.2025.1504614 40260285 PMC12009720

[51] Ren L Li Y Liu S Jia X He H Zhong F . Triple-probiotic-fermented Goji (*Lycium barbarum* L.) ameliorates metabolic disorders associated with hyperuricemia in mice. Microorganisms. (2025) 13:1367. doi: 10.3390/microorganisms13061367 40572255 PMC12196341

[52] Zhang N Zhang B Chen X Zhang Y Wang Y Lu S . Effects and mechanisms of Polygonati Rhizoma polysaccharide on potassium oxonate and hypoxanthine-induced hyperuricemia in mice. Int J Biol Macromol. (2024) 280(Pt 1):135550. doi: 10.1016/j.ijbiomac.2024.135550 39278440

[53] Ramesh D Vijayakumar BG Kannan T. Therapeutic potential of uracil and its derivatives in countering pathogenic and physiological disorders. Eur J Med Chem. (2020) 207:112801. doi: 10.1016/j.ejmech.2020.112801 32927231

[54] Ugbogu EA Schweizer LM Schweizer M. Contribution of model organisms to investigating the far-reaching consequences of PRPP metabolism on human health and well-being. Cells. (2022) 11:1909. doi: 10.3390/cells11121909 35741038 PMC9221600

[55] Yu Q Tan W Ma X Xu L Lin G Cheng J . Prolonged fructose overconsumption activates original biosynthetic and metabolic pathways of endogenous purine in rats. Mol Nutr Food Res. (2025) 69:e70122. doi: 10.1002/mnfr.70122 40442934

[56] Huang Y Deng M Chen Y Cai W Liu N Wan Y . Transcriptomic reveals the effect of tannic acid stress on the cellular structure and growth of Saccharomyces cerevisiae. Food Biosci. (2024) 62:105372. doi: 10.1016/j.fbio.2024.105372

