# Supplementary material for: Guiling prescription attenuates hyperuricemia via multi-target regulation of uric acid metabolism, renal protection, and inflammation: insights from metabolomics and network pharmacology
Source: Front Nutr. 2026 Jan 26;12:1738623. doi: 10.3389/fnut.2025.1738623 (PMC12883377; doi:10.3389/fnut.2025.1738623)
Supplement: Supplementary file 1 [file Supplementary_file_1.docx]

Supplementary Material

Guiling Prescription attenuates Hyperuricemia via Multi-target regulation of uric acid metabolism, renal protection, and inflammation: Insights from Metabolomics and Network Pharmacology

YuKun Wang^1,2,3^, RenJie Ding^1,2,3^, Yaxuan Guo ^1,2,3^, TianHui Zhou ^1,2,3^, Huichun Zhao^4^, HuiWu Liu ^4^, XueMei Qin^1,2,3^, XiaoXia Gao ^1,2,3^*

^1^ Modern Research Center for Traditional Chinese Medicine, Shanxi University, Taiyuan 030006, Shanxi, China

^2^ The Key Laboratory of Chemical Biology and Molecular Engineering of Ministry of Education, Shanxi University, Taiyuan 030006, Shanxi, China

^3^ Key Laboratory of Effective Substances Research and Utilization in TCM of Shanxi Province, Shanxi University, Taiyuan 030006, Shanxi, China

^4^ Shanxi Guangyuyuan Traditional Chinese Medicine, Jinzhong, 030800, Shanxi, China

***Correspondence:**

Corresponding Author: XiaoXia Gao. gaoxiaoxia@sxu.edu.cn

# Chemical composition analysis of GP based on UPLC-Q-TOF-MS analysis

The analysis was conducted with a Waters ACQUITY UPLC® HSS T3 column (2.1 × 100 mm, 1.8 μm) at a flow rate of 0.3 mL/min and a column temperature of 40°C.

A gradient elution consists of 0.1% formic acid-water (A) and formic acid-acetonitrile (B), and the gradient program was set as follows: 0–3 min, 5–15% B; 3–8 min, 15–25% B; 8–13 min, 25–35% B; 13–16 min, 35–60% B; 16–19 min, 60–95% B; 19–20 min, 95–5% B; 20–22 min, 5–5% B. The injection volume was 5 μL.

For the MS analysis, Mass spectrometric analysis was performed with a Q-TOF-MS (Triple TOF 5600+, SCIEX, Foster City, CA, USA) operating in the positive and negative ion mode using an electronic spray ionization (ESI) ion source. The detection mode was set as information dependent acquisition (IDA). For other parameters, the source temperature was 500℃; curtain gas (CUR), ion source GS1 and ion source GS2 were maintained at 30, 55 and 50 psi, respectively. The ion spray voltage was fixed at 5500 V for the positive mode and -4500 V for the negative mode; DP was set at 60 V for the positive mode and -60 V for the negative mode. And the collision energy (CE) was set to 40 eV with a CE spread (CEs) of 20 eV. SCIEX OS-Q 1.6 software was used to collect and process data.


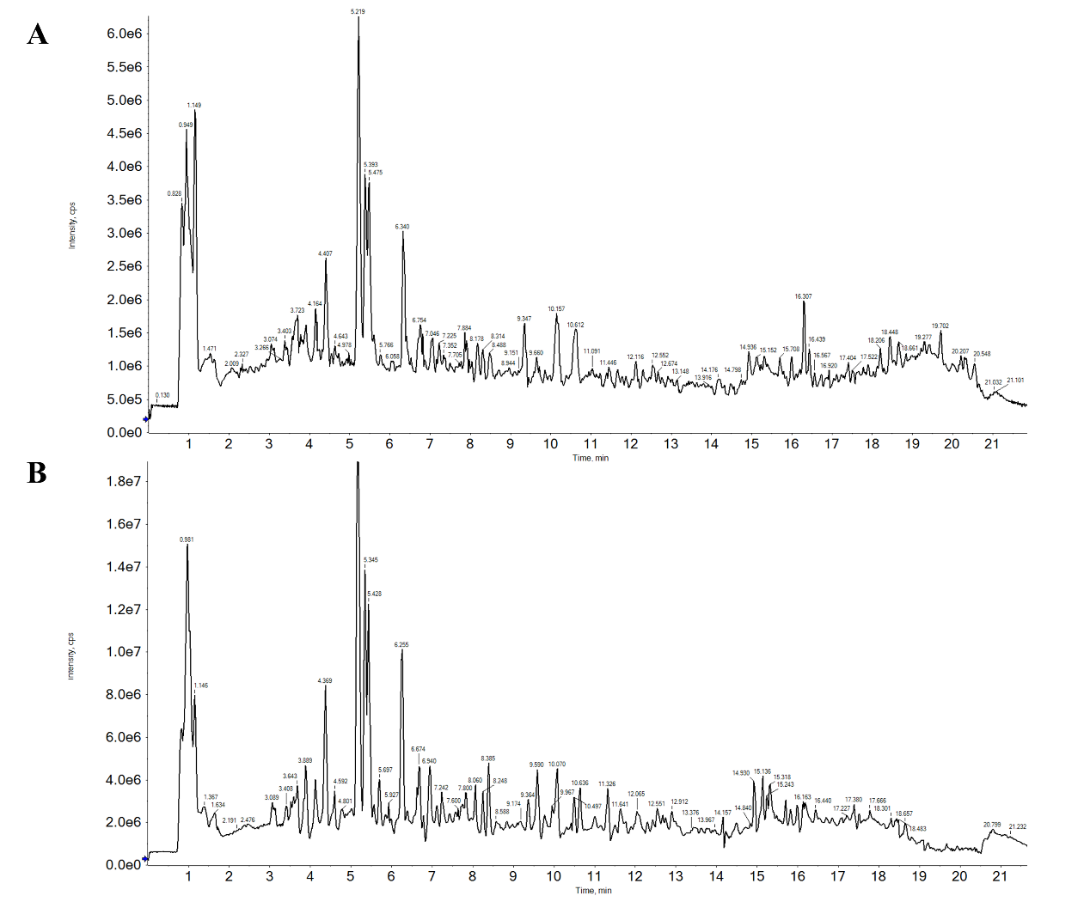


**Supplementary Figure. 1**. Total ion chromatograms (TIC) chromatograms of GP obtained by UPLC-Q-TOF-MS/MS analysis.

A: positive-ion scan, and B: negative-ion scan.

# Metabolomics Analysis Based on UHPLC-Q Exactive Orbitrap-MS

Chromatographic separation was performed on a Waters ACQUITY UPLC HSS T3 (2.1 × 100 mm, 1.8 μm) maintained at 40 °C. The injection volume was 5 μL and the flow rate was 0.2 mL/min. The mobile phase consisted of 0.1% formic acid in water (A) and 0.1% formic acid in acetonitrile (B), the gradient elution conditions were operated under the following program: 0–2 min, 2% B; 2–3 min, 2–35% B; 3–17 min, 35–70% B; 17–18 min, 70% B; 18–29 min, 70–98% B; 29–31 min, 98% B; 31–33 min, 98%–2% B; 33–35 min, 2% B.

Both the positive and negative ion modes of mass detection were set as follows: the heater temperature was 300 °C; the capillary temperature was set as 320 °C; the auxiliary gas flow rate was set as 10 arbs; the sheath gas velocity was 35 arb and the scan range was from m/z 100 to 1500. In addition, an electrospray ionization source was used. Quality control samples (QC) were prepared by mixing the same volume from all supernatant samples (10 μL from each sample), and run randomly throughout the analytical process to check the stability of the system.


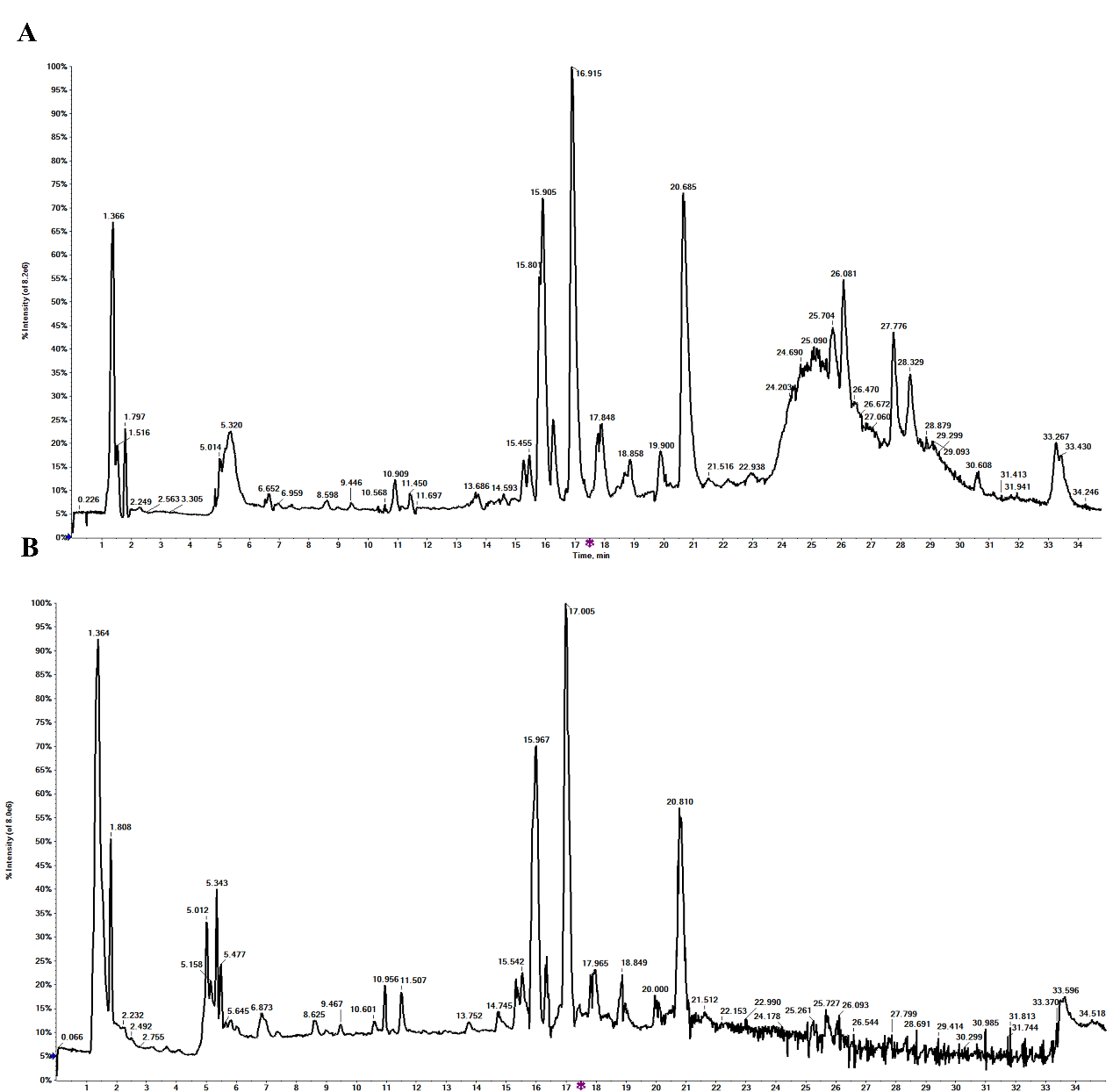


**Supplementary Figure. 2** Total ion chromatograms (TIC) of rat serum samples in positive ion mode (A) and negative ion mode (B)


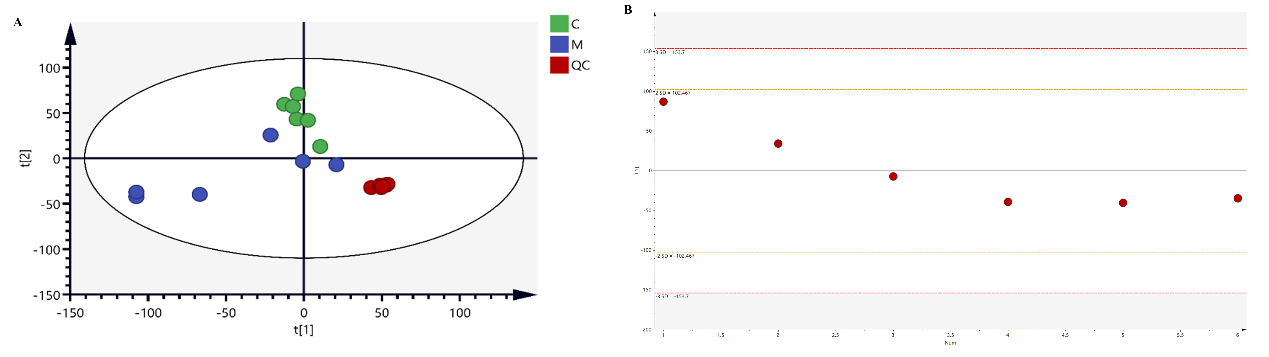


**Supplementary Figure. 3** The PCA score spots of QC samples


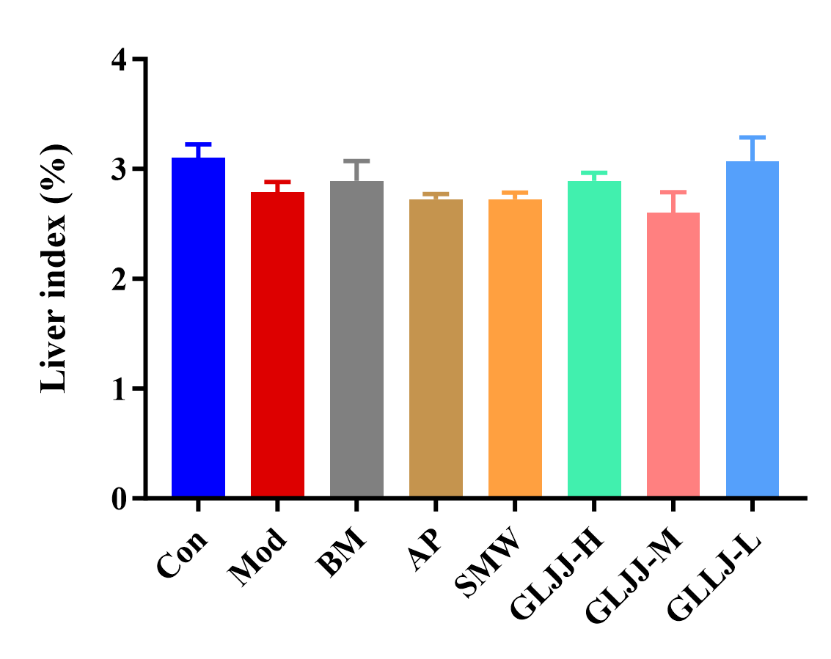


**Supplementary Figure. 4** Liver index. Data are presented as mean ± S.E.M (n = 8).

**Supplementary Table 1** The MS results of constituents identified in the GP

| NO. | Compound name | RT (min) | ION | Formula | Precursor Mass | Found At Mass | Mass Error (ppm) |
| --- | --- | --- | --- | --- | --- | --- | --- |
| 1 | L-Arginine | 0.87 | [M + H] ^+^ | C_6_H_14_N_4_O_2_ | 175.119 | 175.118 7 | -1.2 |
| 2 | D-Galactose | 0.93 | [M - H] ^-^ | C_6_H_12_O_6_ | 179.056 | 179.056 8 | 3.8 |
| 3 | Betaine | 0.95 | [M + H] ^+^ | C_5_H_11_NO_2_ | 118.086 | 118.086 6 | 2.5 |
| 4 | Proline | 0.98 | [M + H] ^+^ | C_5_H_9_NO_2_ | 116.071 | 116.070 9 | 2.4 |
| 5 | Hydroxycitric acid | 1.05 | [M - H] ^-^ | C_6_H_8_O_8_ | 207.015 | 207.015 2 | 2.5 |
| 6 | Adenine | 1.06 | [M + H] ^+^ | C_5_H_5_N_5_ | 136.062 | 136.061 5 | -1.6 |
| 7 | Leucine | 1.15 | [M + H] ^+^ | C_6_H_13_NO_2_ | 132.102 | 132.102 2 | 2 |
| 8 | Citric acid | 1.16 | [M - H] ^-^ | C_6_H_8_O_7_ | 191.02 | 191.020 6 | 4.4 |
| 9 | Adenosine | 1.57 | [M + H] ^+^ | C_10_H_13_N_5_O_4_ | 268.104 | 268.104 3 | 0.9 |
| 10 | Succinic acid | 1.65 | [M - H] ^-^ | C_4_H_6_O_4_ | 117.019 | 117.019 9 | 5.1 |
| 11 | Isoleucine | 1.65 | [M + H] ^+^ | C_6_H_13_NO_2_ | 132.102 | 132.102 1 | 1.1 |
| 12 | Guanosine | 1.67 | [M + H] ^+^ | C_10_H_13_N_5_O_5_ | 284.099 | 284.099 2 | 0.8 |
| 13 | Phenprobamate | 2.56 | [M + H] ^+^ | C_9_H_11_NO_2_ | 166.086 | 166.086 2 | -0.5 |
| 14 | 5-Hydroxymethylfurfural | 2.82 | [M + H] ^+^ | C_6_H_6_O_3_ | 127.039 | 127.039 | 0.6 |
| 15 | Camphor | 3.23 | [M + H] ^+^ | C_8_H_8_O_3_ | 153.055 | 153.054 5 | -0.5 |
| 16 | Glucosylvitexin | 3.52 | [M + H] ^+^ | C_27_H_30_O_15_ | 595.166 | 595.165 3 | -0.8 |
| 17 | Spinosin | 3.90 | [M + H] ^+^ | C_28_H_32_O_15_ | 609.181 | 609.181 | -0.6 |
| 18 | Homoorientin (Isoorientin) | 4.16 | [M + H] ^+^ | C_21_H_20_O_11_ | 449.108 | 449.108 5 | 1.4 |
| 19 | Ligustrazine | 4.33 | [M + H] ^+^ | C_8_H_12_N_2_ | 137.107 | 137.107 7 | 3 |
| 20 | Pueraria glycoside | 4.41 | [M + H] ^+^ | C_21_H_20_O_10_ | 433.113 | 433.113 | 0.2 |
| 21 | Protocatechuic Aldehyde | 4.44 | [M - H] ^-^ | C_7_H_6_O_3_ | 137.024 | 137.024 7 | 2.3 |
| 22 | Aempferol-3-O-rutinoside | 4.46 | [M + H] ^+^ | C_27_H_30_O_15_ | 595.166 | 595.165 1 | -1.1 |
| 23 | Vaccarin | 4.54 | [M + H] ^+^ | C_32_H_38_O_19_ | 727.208 | 727.208 8 | 1.1 |
| 24 | Oroxin B | 4.92 | [M + H] ^+^ | C_27_H_30_O_15_ | 595.166 | 595.165 7 | -0.1 |
| 25 | Lonicerin | 5.15 | [M + H] ^+^ | C_27_H_30_O_15_ | 595.166 | 595.167 3 | 2.6 |
| 26 | Puerarin | 5.22 | [M + H] ^+^ | C_21_H_20_O_9_ | 417.118 | 417.118 2 | 0.5 |
| 27 | Caffeic acid | 5.27 | [M - H] ^-^ | C_9_H_8_O_4_ | 179.035 | 179.035 2 | 1.4 |
| 28 | 3'-Methoxy Puerarin | 5.48 | [M + H] ^+^ | C_22_H_22_O_10_ | 447.129 | 447.129 6 | 2.3 |
| 29 | Regaloside A | 5.7 | [M - H] ^-^ | C_18_H_24_O_10_ | 399.13 | 399.129 4 | -0.6 |
| 30 | Magnoflorine | 5.73 | [M + H] ^+^ | C_20_H_23_NO_4_ | 342.17 | 342.170 1 | 0.4 |
| 31 | 4-Hydroxybenzaldehyde | 5.87 | [M - H] ^-^ | C_7_H_6_O_2_ | 121.03 | 121.030 | 3.7 |
| 32 | Isovitexin | 6.03 | [M + H] ^+^ | C_21_H_20_O_10_ | 433.113 | 433.113 4 | 1.2 |
| 33 | Chrysophanol | 6.26 | [M - H] ^-^ | C_15_H_10_O_4_ | 253.0514 | 253.051 4 | 3.1 |
| 34 | Daidzin | 6.26 | [M - H] ^-^ | C_21_H_20_O_9_ | 415.103 | 415.103 6 | 0.2 |
| 35 | Daidzein | 6.34 | [M + H] ^+^ | C_15_H_10_O_4_ | 255.065 | 255.065 | -0.7 |
| 36 | Calycosin-7-O-glucoside | 6.70 | [M + H] ^+^ | C_22_H_22_O_10_ | 447.129 | 447.129 3 | 1.7 |
| 37 | Luteoloside | 6.8 | [M - H] ^-^ | C_21_H_20_O_11_ | 447.093 | 447.092 8 | -1.1 |
| 38 | Vitexin | 6.88 | [M + H] ^+^ | C_21_H_20_O_10_ | 433.113 | 433.113 2 | 0.7 |
| 39 | Kaempferol | 6.90 | [M + H] ^+^ | C_15_H_10_O_6_ | 287.055 | 287.055 4 | 1.4 |
| 40 | Vicenin 2 | 6.99 | [M + H] ^+^ | C_27_H_30_O_15_ | 595.166 | 595.165 9 | 0.2 |
| 41 | Syringaldehyde | 7.28 | [M + H] ^+^ | C_9_H_10_O_4_ | 183.065 | 183.064 4 | -4.6 |
| 42 | Isoliquiritigenin | 7.40 | [M + H] ^+^ | C_15_H_12_O_4_ | 257.081 | 257.081 1 | 0.9 |
| 43 | Rutin | 7.41 | [M + H] ^+^ | C_27_H_30_O_16_ | 611.161 | 611.160 2 | -0.7 |
| 44 | Tectoridin | 7.57 | [M - H] ^-^ | C_21_H_18_O_12_ | 461.073 | 461.072 6 | 0.2 |
| 45 | Isoscopoletin | 7.64 | [M + H] ^+^ | C_10_H_8_O_4_ | 193.05 | 193.049 5 | -0.3 |
| 46 | Scutellarin | 7.74 | [M - H] ^-^ | C_21_H_18_O_12_ | 461.073 | 461.072 1 | -1 |
| 47 | Scutellarein | 7.76 | [M + H] ^+^ | C_15_H_10_O_6_ | 287.055 | 287.055 4 | 0.7 |
| 48 | Luteolin-7-O-β-D-glucuronide | 7.85 | [M + H] ^+^ | C_21_H_18_O_12_ | 463.087 | 463.087 3 | 0.4 |
| 49 | Sophoricoside | 8.07 | [M - H] ^-^ | C_21_H_20_O_10_ | 431.098 | 431.097 8 | -1.3 |
| 50 | Emodin | 8.16 | [M + H] ^+^ | C_15_H_10_O_5_ | 271.06 | 271.060 | -0.3 |
| 51 | Apigenin-7-glucoside | 8.17 | [M + H] ^+^ | C_21_H_20_O_10_ | 433.113 | 433.113 7 | 1.7 |
| 52 | Baicalin | 9.18 | [M-H]- | C_21_H_18_O_11_ | 445.078 | 445.077 4 | -0.5 |
| 53 | Apigenin 7-O-beta-D-glucuronide | 9.30 | [M + H] ^+^ | C_21_H_18_O_11_ | 447.092 | 447.093 1 | 2.1 |
| 54 | 4-Hydroxybenzoic acid | 9.35 | [M - H] ^-^ | C_7_H_6_O_3_ | 137.024 | 137.024 7 | 2.3 |
| 55 | Rosmarinic acid | 9.6 | [M - H] ^-^ | C_18_H_16_O_8_ | 359.077 | 359.077 1 | -0.5 |
| 56 | Ferulic Acid | 9.76 | [M - H] ^-^ | C_10_H_10_O_4_ | 193.051 | 193.050 7 | 0.6 |
| 57 | Isoferulic acid | 9.97 | [M - H] ^-^ | C_10_H_10_O_4_ | 193.051 | 193.050 7 | 0.6 |
| 58 | Ononin | 10.57 | [M + H] ^+^ | C_22_H_22_O_9_ | 431.134 | 431.133 5 | -0.4 |
| 59 | Ginsenoside Rg1 | 10.63 | [M + HCOO] ^-^ | C_42_H_72_O_14_ | 845.49 | 845.492 2 | 2.1 |
| 60 | Ginsenoside Rd | 10.65 | [M + HCOO] ^-^ | C_48_H_82_O_18_ | 991.548 | 991.550 8 | 2.5 |
| 61 | Isovanillin | 10.96 | [M + H] ^+^ | C_8_H_8_O_3_ | 153.055 | 153.054 5 | -0.2 |
| 62 | Tectorigenin | 11.18 | [M - H] ^-^ | C_16_H_12_O_6_ | 299.056 | 299.055 8 | -1.1 |
| 63 | Daidzein | 11.32 | [M - H] ^-^ | C_15_H_10_O_4_ | 253.051 | 253.051 4 | 1.6 |
| 64 | Chrysin | 11.45 | [M + H] ^+^ | C_15_H_10_O_4_ | 255.065 | 255.065 | -0.7 |
| 65 | Isoliquiritigenin | 11.63 | [M - H] ^-^ | C_15_H_12_O_4_ | 255.0661 | 255.065 9 | -0.6 |
| 66 | 6''-O-Acetylgenistin | 11.69 | [M - H] ^-^ | C_23_H_22_O_11_ | 473.109 | 473.108 1 | -1.7 |
| 67 | Calycosin | 11.89 | [M + H] ^+^ | C_16_H_12_O_5_ | 285.076 | 285.075 9 | 0.4 |
| 68 | Luteolin | 12.2 | [M - H] ^-^ | C_15_H_10_O_6_ | 285.04 | 285.040 6 | 0.6 |
| 69 | Bomyl acetate | 12.25 | [M + H] ^+^ | C_8_H_8_O_2_ | 137.06 | 137.059 7 | 0.1 |
| 70 | Gracillin | 12.54 | [M + H] ^+^ | C_45_H_72_O_17_ | 885.484 | 885.484 7 | 0.6 |
| 71 | Ethyl caffeate | 12.67 | [M - H] ^-^ | C_11_H_12_O_4_ | 207.066 | 207.066 2 | -0.3 |
| 72 | Glycetein | 12.78 | [M + H] ^+^ | C_16_H_12_O_5_ | 285.076 | 285.075 7 | -0.3 |
| 73 | Biochanin A | 13.09 | [M - H] ^-^ | C_16_H_12_O_5_ | 283.061 | 283.061 6 | 1.4 |
| 74 | Pectolinarigenin | 13.1 | [M - H] ^-^ | C_17_H_14_O_6_ | 313.072 | 313.071 6 | -0.4 |
| 75 | Dipsacoside B | 13.91 | [M - H] ^-^ | C_53_H_86_O_22_ | 1119.559 | 1119.561 | 1.9 |
| 76 | Naringenin | 14.08 | [M - H] ^-^ | C_15_H_12_O_5_ | 271.061 | 271.061 3 | 0.2 |
| 77 | Pseuoginsenoside F11 | 14.15 | [M - H] ^-^ | C_42_H_72_O_14_ | 799.485 | 799.485 8 | 1.1 |
| 78 | Purpurin | 14.17 | [M + H] ^+^ | C_14_H_8_O_5_ | 257.044 | 257.044 4 | -0.2 |
| 79 | 3-Hydroxymorindone | 14.23 | [M - H] ^-^ | C_15_H_10_O_6_ | 285.0401 | 285.040 6 | -1.4 |
| 80 | 6,7,4'-Trihydroxyisoflavone | 14.36 | [M + H] ^+^ | C_15_H_10_O_5_ | 271.06 | 271.060 6 | 1.9 |
| 81 | Genistein | 14.36 | [M + H] ^+^ | C_15_H_10_O_5_ | 271.06 | 271.060 6 | 1.9 |
| 82 | Coumestrol | 14.4 | [M - H] ^-^ | C_15_H_8_O_5_ | 267.03 | 267.029 8 | -0.5 |
| 83 | Kaempferide | 14.75 | [M + H] ^+^ | C_16_H_12_O_6_ | 301.071 | 301.071 | 1.1 |
| 84 | 20(R)-Ginsenoside Rg3 | 15.07 | [M + HCOO] ^-^ | C_42_H_72_O_13_ | 829.495 | 829.495 2 | -0.4 |
| 85 | Ginsenoside Rc | 15.15 | [M - H] ^-^ | C_53_H_90_O_22_ | 1077.585 | 1077.585 | -0.5 |
| 86 | 20(R)-Ginsenoside Rh1 | 15.18 | [M + HCOO] ^-^ | C_36_H_62_O_9_ | 683.438 | 683.437 1 | -0.8 |
| 87 | 2-Hydroxyadenosine | 15.20 | [M + H] ^+^ | C_10_H_13_N_5_O_5_ | 284.099 | 284.100 3 | 4.9 |
| 88 | Pedunculoside | 15.22 | [M + HCOO] ^-^ | C_36_H_58_O_10_ | 695.401 | 695.399 5 | -2.5 |
| 89 | Ginsenoside-Ro | 15.24 | [M - H] ^-^ | C_48_H_76_O_19_ | 955.491 | 955.491 1 | 0.3 |
| 90 | Ginsenoside Rb2 | 15.31 | [M - H] ^-^ | C_53_H_90_O_22_ | 1077.585 | 1077.586 | 0.7 |
| 91 | Asperosaponin Ⅵ | 15.35 | [M + HCOO] ^-^ | C_47_H_76_O_18_ | 973.501 | 973.501 6 | 0.3 |
| 92 | Madecassoside | 15.35 | [M - H] ^-^ | C_48_H_78_O_20_ | 973.501 | 973.501 6 | 0.3 |
| 93 | 20(S)-Ginsenoside F1 | 15.38 | [M + HCOO] ^-^ | C_36_H_62_O_9_ | 683.438 | 683.437 2 | -0.6 |
| 94 | A 3-N-butyl-4,5-dihydrophthalide | 15.42 | [M + H] ^+^ | C_10_H_8_O_4_ | 193.0486 | 193.048 6 | -4.8 |
| 95 | Ethyl ferulate | 15.71 | [M + H] ^+^ | C_12_H_14_O_4_ | 223.096 | 223.096 3 | -0.9 |
| 96 | Formononetin | 15.88 | [M + H] ^+^ | C_16_H_12_O_4_ | 269.081 | 269.080 9 | 0.2 |
| 97 | Notoginsenoside Fe | 16.26 | [M + HCOO] ^-^ | C_47_H_80_O_17_ | 961.538 | 961.538 4 | 0.6 |
| 98 | Notoginsenoside Ft1 | 16.26 | [M-H]- | C_48_H_82_O_19_ | 961.538 | 961.538 4 | 0.6 |
| 99 | Wogonin | 16.37 | [M + H] ^+^ | C_16_H_12_O_5_ | 285.076 | 285.075 9 | 3.5 |
| 100 | Digitoxin | 16.44 | [M + HCOO] ^-^ | C_41_H_64_O_13_ | 809.433 | 809.433 6 | 0.8 |
| 101 | Madecassic acid | 16.57 | [M - H] ^-^ | C_30_H_48_O_6_ | 503.338 | 503.337 4 | -0.8 |
| 102 | 6-Gingerol | 16.91 | [M + H] ^+^ | C_17_H_26_O_4_ | 295.19 | 295.190 7 | 0.9 |
| 103 | Pinocembrin | 17.04 | [M + H] ^+^ | C_15_H_12_O_4_ | 257.081 | 257.081 1 | 0.8 |
| 104 | Chikusetsusponin IVa | 17.04 | [M - H] ^-^ | C_42_H_66_O_14_ | 793.438 | 793.438 4 | 0.6 |
| 105 | Ganoderic acid C2 | 17.07 | [M - H] ^-^ | C_30_H_46_O_7_ | 517.317 | 517.315 6 | -2.9 |
| 106 | Neobavaisoflavone | 17.13 | [M + H] ^+^ | C_20_H_18_O_4_ | 323.128 | 323.128 | 0.7 |
| 107 | Acacetin | 17.2 | [M - H] ^-^ | C_16_H_12_O_5_ | 283.061 | 283.061 1 | 1.4 |
| 108 | Ecliptasaponin A | 17.27 | [M - H] ^-^ | C_36_H_58_O_9_ | 633.401 | 633.400 3 | -0.9 |
| 109 | Ruscogenin | 17.35 | [M + H] ^+^ | C_27_H_42_O_4_ | 431.316 | 431.315 8 | 0.5 |
| 110 | 20(S)Ginsenoside Rg3 | 17.41 | [M + HCOO] ^-^ | C_42_H_72_O_13_ | 829.495 | 829.495 8 | 0.3 |
| 111 | Ginsenoside Rg2 | 17.44 | [M - H] ^-^ | C_42_H_72_O_13_ | 783.49 | 783.490 5 | 0.6 |
| 112 | Panaxydol | 17.61 | [M + H] ^+^ | C_17_H_24_O_2_ | 261.185 | 261.185 1 | 0.7 |
| 113 | Asiatic acid | 17.75 | [M + H] ^+^ | C_30_H_48_O_5_ | 489.357 | 489.357 | -0.9 |
| 114 | Corylin | 17.87 | [M - H] ^-^ | C_20_H_16_O_4_ | 319.098 | 319.097 1 | -1.5 |
| 115 | Ginsenoside Rf | 17.97 | [M - H] ^-^ | C_42_H_72_O_14_ | 799.485 | 799.484 9 | -0.1 |
| 116 | Gingerglycolipid B | 18.31 | [M + HCOO] ^-^ | C_33_H_58_O_14_ | 723.381 | 723.381 5 | 0.9 |
| 117 | Liriopesides B | 18.45 | [M + HCOO] ^-^ | C_39_H_62_O_12_ | 767.422 | 767.422 | -0.4 |
| 118 | Echinocystic acid | 18.52 | [M - H] ^-^ | C_30_H_48_O_4_ | 471.348 | 471.348 3 | 0.6 |
| 119 | Sclareolide | 18.71 | [M + H] ^+^ | C_16_H_26_O_2_ | 251.201 | 251.201 2 | 2.6 |
| 120 | Methylophiopogonanone B | 18.74 | [M - H] ^-^ | C_19_H_20_O_5_ | 327.124 | 327.123 | -2.5 |
| 121 | 4'-O-Methylbroussochalcone B | 18.76 | [M + H] ^+^ | C_21_H_22_O_4_ | 339.159 | 339.159 6 | 1.4 |
| 122 | α-Cyperone | 18.84 | [M + H] ^+^ | C_15_H_22_O | 219.174 | 219.174 1 | -1 |
| 123 | Hederagenin | 19.17 | [M - H] ^-^ | C_30_H_48_O_4_ | 471.348 | 471.346 7 | -2.7 |
| 124 | β-elemene | 19.96 | [M + H] ^+^ | C_15_H_24_ | 205.195 | 205.195 | -0.3 |
